# How the Electrochemical Double Layer Manipulates Molecule–Metal Interactions

**DOI:** 10.1021/acsnano.6c03605

**Published:** 2026-06-18

**Authors:** Tabitha Jones, Minho M. Kim, Sarah May Sibug-Torres, Elle Wyatt, Nicolas Spiesshofer, James W. Beattie, Jonathan Bar-David, Rakesh Arul, Bart de Nijs, Hyungjun Kim, Jeremy J. Baumberg

**Affiliations:** † NanoPhotonics Centre, Cavendish Laboratory, Department of Physics, University of Cambridge, JJ Thompson Avenue, Cambridge CB3 0HE, United Kingdom; ‡ Department of Chemistry, 34968 Korea Advanced Institute of Science and Technology, Daejeon 34141, Republic of Korea; § Physics for Sustainable Chemistry Group, Cavendish Laboratory, Department of Physics, University of Cambridge, JJ Thompson Avenue, Cambridge CB3 0HE, United Kingdom

**Keywords:** surface-enhanced Raman, cyclic voltammetry, electrochemical double layer, sensing, nucleobases

## Abstract

Electrochemical interfaces are ubiquitous in sensing, catalysis, and energy storage, yet understanding molecular interactions with the electrochemical double layer (EDL) remains limited. Here, we use electrochemical surface-enhanced Raman spectroscopy (EC-SERS) to probe analyte–EDL interactions in real time. Precision SERS electrodes with robust electrochemically recleanable gold nanogaps allow us to detect subtle molecular spectral changes during cyclic voltammetry, revealing distinct intensity and frequency oscillations. Quantum mechanics/molecular mechanics simulations show that these effects arise from electrochemical potential-induced molecular reorientation and surface restructuring driven by dynamic interactions with the EDL. For sensing, this mechanism reduces detection limits for DNA nucleobases by more than 25-fold and enables label-free multiplexed sensing. Beyond improved sensor performance, this work provides a framework for understanding EC-SERS and gives insight into neutral molecule behavior within the EDL.

## Graphical abstract

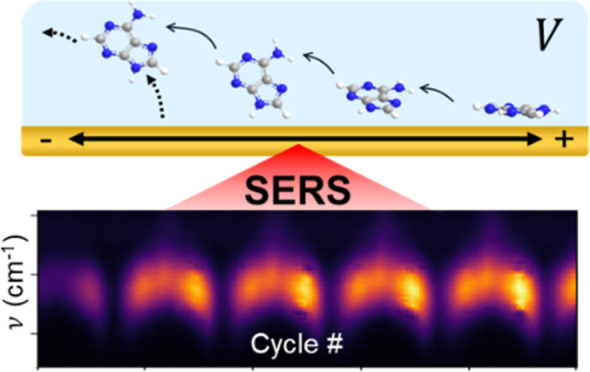

Understanding how molecules behave at electrochemical interfaces is crucial for progress in sensing, catalysis, energy storage, and molecular electronics. Central to this is the electrochemical double layer (EDL), the nanoscale interfacial region between a charged electrode and a liquid electrolyte.^1,2^ Classical models, such as Gouy–Chapman–Stern, describe the EDL as a static arrangement of compact and diffuse ionic layers that screen surface charge.^3,4^ However, these models typically neglect how analytes interact with the EDL and fail to capture the dynamic nature of molecular behavior at the interface.^5,6^

Despite its importance, the EDL remains extremely challenging to study. Comprising ions, solvent, analytes, and the electrode surface, it is a highly coupled system buried at the interface between two bulk phases, making it difficult to isolate and resolve at the molecular level.^1,5,7^ Studies using X-ray photoelectron spectroscopy,^8^ X-ray absorption spectroscopy,^9^ and shell-isolated nanoparticle-enhanced Raman spectroscopy (SHINERS)^10^ have made significant strides in understanding the behavior of interfacial water in the EDL. However, the interactions between neutral molecular analytes and the EDL have received comparatively little attention. Surface-enhanced Raman spectroscopy (SERS) is a powerful tool for studying molecules at metal surfaces, offering detailed insights into molecular structure, orientation, and surface interactions even at trace concentrations.^11^ Electrochemical-SERS (EC-SERS) extends this capability by incorporating the SERS substrate into an electrochemical cell, allowing spectra to be recorded under an applied potential.^12,13^ EC-SERS has been employed since the earliest SERS demonstrations,^14^ with studies observing potential-dependent changes in spectral intensity and peak position for adsorbed molecules such as pyridine. These changes have been variously attributed to molecular reorientation, charge-transfer resonances, and changes in surface coverage.^15–17^ Complementary electrochemical measurements on single-crystal electrodes have studied potential-dependent molecular reorientation more directly,^18,19^ but these flat surfaces do not provide the nanoscale features required for SERS enhancement.

Conversely, mechanistic insights from SERS have been limited by heterogeneous substrates with ill-defined geometries that obscure subtle spectral changes.^20^ Furthermore, EC-SERS measurements have typically been performed at fixed potentials after equilibration, preventing the study of dynamic molecular behavior. These limitations, combined with the absence of simulations capable of capturing the full complexity of analyte–ion–solvent interactions at interfaces, have hindered mechanistic understanding. Recent work has advanced the understanding of an interfacial water structure^10,21^ and the spectro-electrochemical response of redox-active molecules,^22,23^ but a molecular-level picture of how neutral analytes interact with the EDL remains elusive.

Recently, highly reproducible and reusable thin-film SERS substrates have become available from bottom-up self-assembly of AuNPs using a rigid barrel-shaped molecular scaffold, cucurbit­[*n*]­uril (CB­[*n*], *n* = 5–8), which yield precisely identical nanogaps.^24,25^ These substrates can be reliably precleaned, recleaned, and regenerated using successive electrochemical oxidation and reduction steps.^26^ Such reproducible substrates allow us to carefully isolate and identify the effects of applied potential, probing the behavior of analytes and the EDL within nanogaps.

We first study the EC-SERS behavior of exemplar molecules, neutral DNA nucleobases. Despite prior work,^27–29^ there is still debate over the nature of their Au surface adsorption^28,30–32^ and EC-SERS behavior.^33^ We show that by cycling the electrochemical potential, we can significantly increase the SERS from nucleobases, dramatically reducing their limits of detection (LoDs) by over 25×. For each analyte, we observe repeatable oscillations in the intensity and position of their characteristic vibrational peaks, which result from the potential-induced molecular reorientation and molecular interaction with the EDL. Understanding of this mechanism is supported by first-principle-based molecular simulations that reveal how cations dynamically coordinate with analytes, resulting in a highly structured EDL shell. These findings provide a molecular-level explanation for the EC-SERS enhancement and enable the simultaneous multiplexed detection of all four nucleobases, facilitating label-free biomarker detection in complex fluids. More broadly, this work establishes a framework for understanding analyte–EDL interactions and demonstrates the potential of EC-SERS for mechanistic studies of electrochemical interfaces.

## Results and Discussion

To fabricate reproducible EC-SERS substrates with precise sub-1 nm nanogaps, we self-assemble citrate-stabilized 80 nm AuNPs using CB[5], which binds them through its carbonyl portals.^34^ The AuNP/CB[5] assembly is concentrated and deposited as a thin film onto conductive FTO-coated glass (Figure S1).^24^ These close-packed near-monolayer AuNP aggregates (termed “MLaggs”) are used as the working electrode in a spectro-electrochemical cell (Figure 1a).

**Figure 1:**
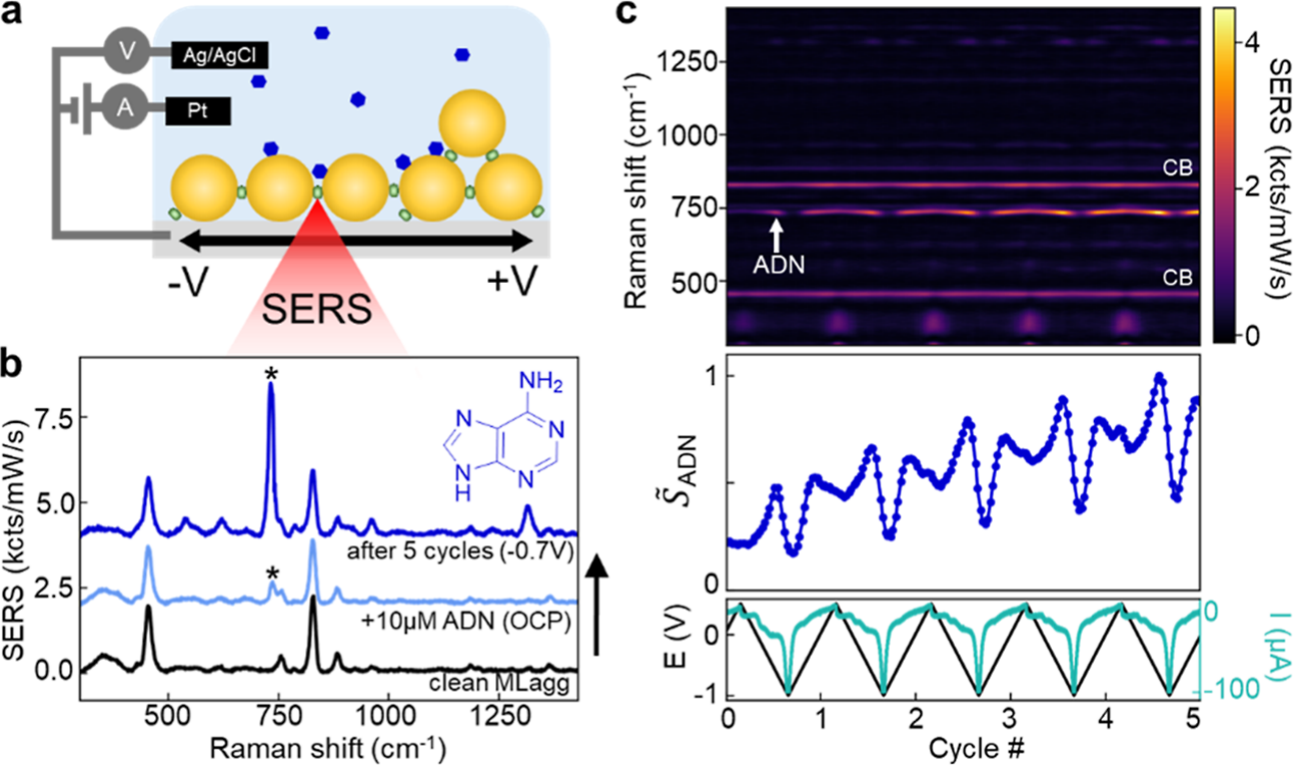
Cyclic electrochemical SERS of adenine using an MLagg substrate. (**a**) 80 nm AuNPs self-assembled with cucurbit[5]­uril (CB[5], green) and deposited onto FTO-coated glass substrate. Spectro-electrochemical cell consists of the MLagg-CB[5] substrate (working electrode), Pt wire (counter electrode), and Ag/AgCl (reference electrode), with analytes (blue hexagons) in an aqueous solution. (**b**) SERS spectra of MLagg after initial in-situ electrochemical cleaning and regeneration (black line), after the addition of 10 μM ADN at open-circuit potential (OCP, light blue), and after 5 EC cycles (recorded at −0.7 V, blue). The ADN peak at 732 cm^–1^ is indicated by *, and the inset shows neutral ADN (dominant at pH 7.0). (**c**) Time-series SERS spectra (1 s integration time, 785 nm 1 mW laser) when MLagg cycled between +0.5 V and −1 V in 10 μM ADN and 50 mM potassium phosphate buffer (pH 7.0) at 50 mV s^–1^ for 5 cycles. Normalized peak area for ADN peak ${\mathrm{S̃}}_{ADN}$ at 732 cm^–1^ (blue), applied potential (black), and current (cyan) plotted vs cycle.

We reliably clean and regenerate the MLaggs using a recently developed in-situ electrochemical SERS nanogap regeneration scheme (“ReSERS”).^26^ First, the applied potential is stepped to +1.5 V to strip the ligand-stabilized nanogaps of molecular adsorbates and form a metastable gold oxide plug. Then, the nanogaps are regenerated by stepping the potential to −0.8 V in a solution containing the CB[5] scaffolding molecule, which reduces the oxide layer and restabilizes the nanogaps. This cleaning procedure can be repeated >100 times without damaging the substrate, producing reliable EC-SERS substrates (<5% relative standard deviation) with large enhancement factors (>10^6^), enabling us to carefully study the electrochemical behavior of analytes at gold surfaces.^26^

Adenine (ADN) is chosen as a model analyte because it has been extensively studied.^27–29^ SERS spectra are recorded by illuminating the MLagg with a 785 nm laser through the transparent FTO-coated glass. Spectra of the precleaned MLaggs recorded at open-circuit potential (OCP ∼0.2 V vs Ag/AgCl) in 50 mM potassium phosphate buffer (KPB, pH 7.0) show two characteristic CB[5] peaks at 456, 828 cm^–1^ (Figure 1b).^26^ When 10 μM ADN is added, a small peak (*) is observed at 732 cm^–1^, assigned to the symmetric ring breathing mode coupled with its in-plane NH_2_ bend (Figure 1b).^27^ At pH 7.0, ADN is expected to be neutral and is only deprotonated at its p*K*_a_ of 9.8.

We perform cyclic voltammetry (without Au oxidation) in 10 μM ADN and 50 mM KPB solution while recording the SERS of the MLagg. The potential is swept from 0 V to +0.5 V, reversed to −1 V, and then back to 0 V at 50 mV s^–1^ for 5 cycles. In the MLagg EC-SERS spectra, significant changes are observed for the ADN peaks, particularly at 732 cm^–1^ (ν_ADN_), where the intensity increases more than 400% in 5 cycles (Figure 1c), before plateauing after ∼5 cycles (Figure S4). At the same time, small shifts in the CB[5] peaks (<2 cm^–1^) are seen, with a ∼30% intensity increase at positive potentials (Figures S5 and S6). Very weak peaks at 350, 555 cm^–1^ associated with the oxidation of gold^26^ also start to appear above +0.25 V (Figure S7).

Consistent oscillations in the magnitude and position of ν_ADN_ (Figure 2a) are seen during EC cycling. In the first EC cycle, the potential is scanned from 0 V to +0.5 V and then to −1 V. During the initial positive scan, only minor changes are observed in ν_ADN_. However, as the potential is swept negatively, the intensity of ν_ADN_ increases, reaching a maximum at ∼−0.6 V, before dropping sharply at −1 V.

**Figure 2:**
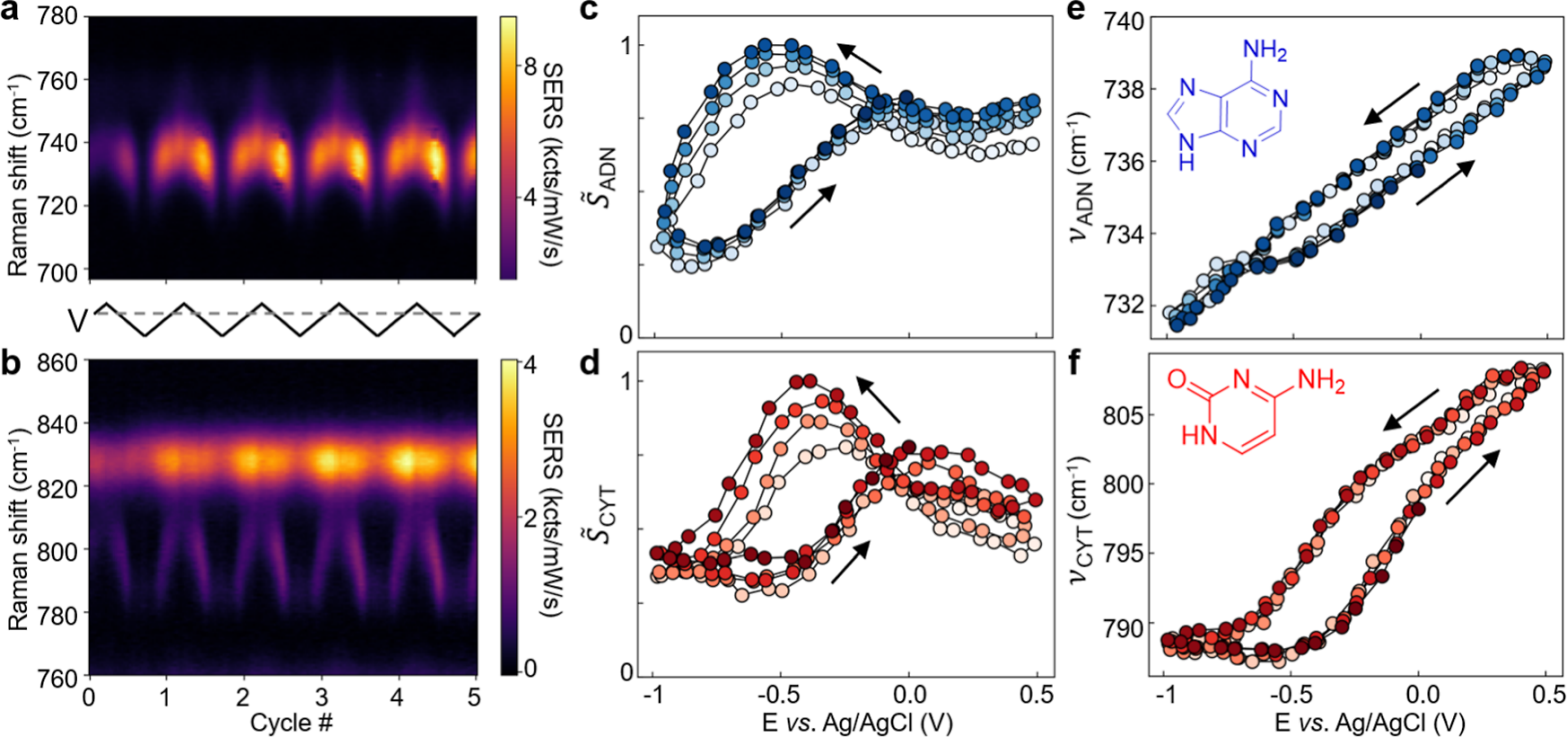
EC-SERS response of adenine vs cytosine. (**a,b**) Time-series SERS spectra of (a) 10 μM adenine (ADN) and (b) 10 μM cytosine (CYT), conditions and cycling as shown in Figure 1. (**c,d**) Normalized SERS peak area vs applied potential (vs Ag/AgCl) for ring-breathing modes of (c) ADN at 732 cm^–1^ and (d) CYT at 796 cm^–1^. (**e,f**) Peak frequency vs applied potential for peaks in (c,d). Marker color hue denotes the time elapsed, and arrows indicate cycle direction. The first cycle is removed for clarity.

From the second cycle onward, a reproducible pattern is observed: during the positive scan from −1 V, the intensity of ν_ADN_ increases (Figure 2c) and the peak shifts toward higher wavenumbers (Figure 2e). The intensity of ν_ADN_ reaches a maximum ∼−0.1 V and then plateaus while continuing to spectrally shift, reaching 739 cm^–1^ at +0.5 V. During the negative scan, the intensity increases again, reaching a new larger maximum at *V*_max_ = −0.6 V. As the negative scan proceeds to −1 V, the peak intensity dramatically reduces, and the peak returns to 732 cm^–1^. These positive and negative scans show a clear hysteresis in both the intensity and position of ν_ADN_. This hysteresis reduces when the scan rate is reduced 10-fold to 5 mV/s (Figures S8–S10). These observations are key to understanding the EC cycling enhancement.

The 4-fold enhancement in ν_ADN_ observed over 5 EC cycles cannot be achieved by simply applying a constant potential for 5 min (or longer) (Figure S11). While many EC-SERS studies focus on identifying a single optimal potential for detection,^12,35,36^ cycling the potential for 5 min results in peaks that are >200% of those recorded after applying a constant potential (−0.7 V) for the same time (Figure S11). Increasing the scan rate to 500 mV/s accelerates this process, achieving the same signal >10× faster than applying a constant potential (Figure S11).

Performing the same cycling with cytosine (CYT), another nucleobase, reveals similar behavior (Figures 2b, S12, and S13). Its characteristic peak at 796 cm^–1^ (ν_CYT_, ring breathing mode^27^) also increases over 5 cycles with hysteretic oscillations in magnitude and tuning within each cycle (Figure 2d,f). However, consistent differences are observed, with the negative scan signal maxima in particular shifting from *V*_max_^ADN^ = −0.6 V for ADN to −0.4 V for CYT (Figure 2c,d). Potential cycling thus enables selective enhancement of analyte specific peaks.

Simulations can help explain the EC-SERS behavior (Figure 3). Besides shifts in the primary ADN and CYT peaks, oscillations are also observed in other characteristic peaks, with each responding differently to applied potential (Figure 3b,c). For ADN, the intensities for 965 cm^–1^ (5-ring deformation), 1318 cm^–1^ (C–N stretch), and 1360 cm^–1^ (N–C–H in-plane bending) modes are maximized at *V*_max_ = +0.5 V, −0.9 V, and +0.2 V, respectively (Figure 3c). While vibrational Stark effects, where molecular vibrational energies are modulated by an external electric field, can account for some changes in SERS intensity and peak position, previously reported shifts (∼3 cm^–1^) for nitrile-containing self-assembled monolayers are modest compared to >15 cm^–1^ shifts observed here.^37,38^ These oscillations also do not align with deprotonation mechanisms (p*K*_a_ is uncorrelated with *V*_max_, see Figure S14). Furthermore, photoinduced charge transfer is ruled out by the observation that comparable enhancement is achieved when potential cycling is performed without laser illumination (Figure S15), while charge transfer is excluded as identical spectroscopic signatures are seen for SERS using a 633 nm laser. This instead suggests EDL-driven reorientation that aligns different bonds with the perpendicular electrical field at different potentials. In a simple picture, the permanent dipole **p** of neutral solvated aromatic analytes reduces their energy (U = -**p**.**E**) when aligned parallel to the applied electric field **E**, eventually overcoming the van der Waals attraction of the flat face of the aromatic molecule to the Au facet (Figure 3a). To understand this in detail, we utilize a recently developed^39^ mean-field quantum-mechanics/molecular-mechanics (QM/MM) simulation to accurately model nucleobases, ions, and water near the electrode surface at different applied potentials (which is extremely challenging, see Methods).^39^ These simulations confirm that nucleobases lie flat on the Au surface at OCP (Figure 3e) but reorient vertically as the potential is swept negative (Figure 3f). As Raman scattering is proportional to the fourth power of the polarizability component in the direction of the optical field (perpendicular to the Au surface), molecular reorientation results in large SERS intensity changes (providing the polarizability tensor of the vibrational mode is anisotropic).^40,41^ The potential at which simulations indicate a majority of ADN molecules adopt a vertical orientation (Figure 3d, −0.6 V) closely matches *V*_max_^ADN^ for the in-plane 732 cm^–1^ ring-breathing mode observed in EC-SERS measurements (Figure 2b), consistent with the SERS surface selection rules.^41^ In contrast, CB[5] is rigidly bound and cannot reorient and consequently shows minimal spectral shifts (<2 cm^–1^, Figure S6). A key insight from the molecular simulations is the dynamic interaction between analyte molecules and K^+^ ions (Figure S16). While the EDL is often treated as a rigid homogeneous structure, our simulations reveal that K^+^ ions actively coordinate with the nucleobases, effectively “dressing” the molecules and stabilizing their vertical orientation under negative potentials (Figure 3f). This ion-mediated stabilization is further supported by cyclic EC-SERS measurements in different concentrations of KPB (pH 7.0) (Figure S17). We observe a consistent increase in SERS intensity with buffer concentration, with a ∼300% enhancement from 5 mM to 500 mM KPB after five cycles (Figure S17), highlighting the critical role of buffer ions in supporting molecular reorientation. A crucial advance here is thus to reveal that the interaction between analytes and the EDL is spatially highly structured.

**Figure 3:**
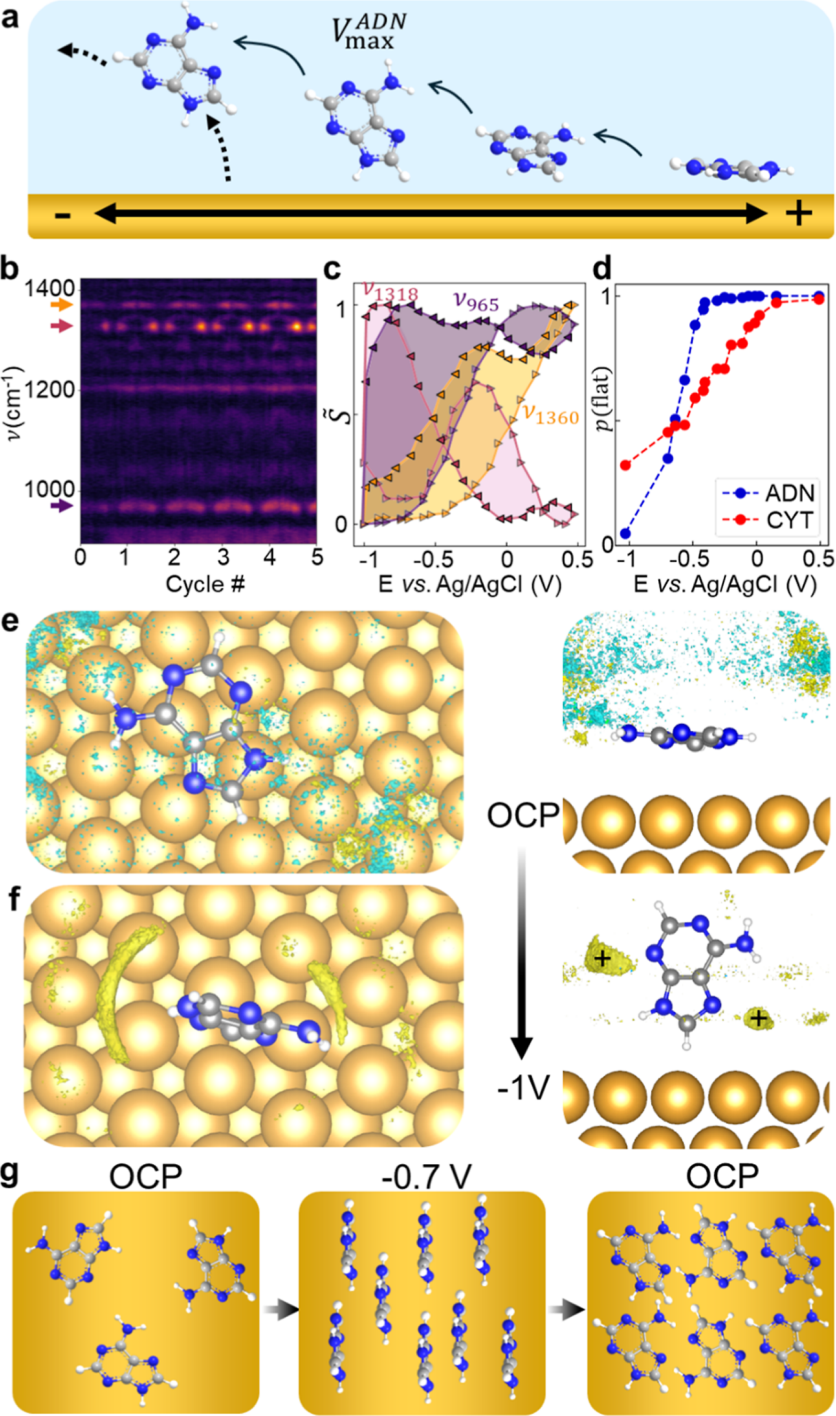
Proposed mechanism. (**a**) Schematic potential-induced molecular reorientation and desorption of adenine from Au facet. (**b**) Time-series SERS spectra of 10 μM ADN, conditions and cycling as shown in Figure 1, arrows mark three extra ADN peaks at 965 cm^–1^ (purple), 1318 cm^–1^ (pink), and 1360 cm ^–1^ (orange). (**c**) Normalized intensities vs potential on cycle 5 for these ADN peaks (scanning positive = ▶, negative = ◀). (**d**) Simulated probability of flat orientation p­(flat) for ADN (blue) and CYT (red) vs potential. (**e,f**) Representative snapshots of the simulated systems of ADN in 50 mM KPB (KPB) at (e) near OCP (0.16 V) and (f) −1.0 V. Charge accumulation and depletion are shown in yellow and cyan, respectively, at ± 0.003 e Å^–3^ (e) and 0.001 e Å^–3^ (f) isosurface level. (**g**) Improved molecular packing after electrochemical cycling.

Different nucleobases exhibit distinct EC-SERS oscillations (Figure 2). QM/MM simulations indeed reveal that cytosine reorients at less negative potentials than adenine (Figures 3d and S18), correlating with the experiment. This threshold is set by the balance between adsorption when flat and alignment of **p** with the interfacial electric field. Our model is supported by previous thermal desorption^42,43^ and computational studies,^32,44,45^ which report a consistent trend in flat adsorption energies (G > A > C > T) that mirrors the sequence of reorientation potentials observed (Figures 2 and 4e). These differences are attributed to variations in π-conjugation between purines and pyrimidines and the binding affinity of their anchoring groups.

**Figure 4:**
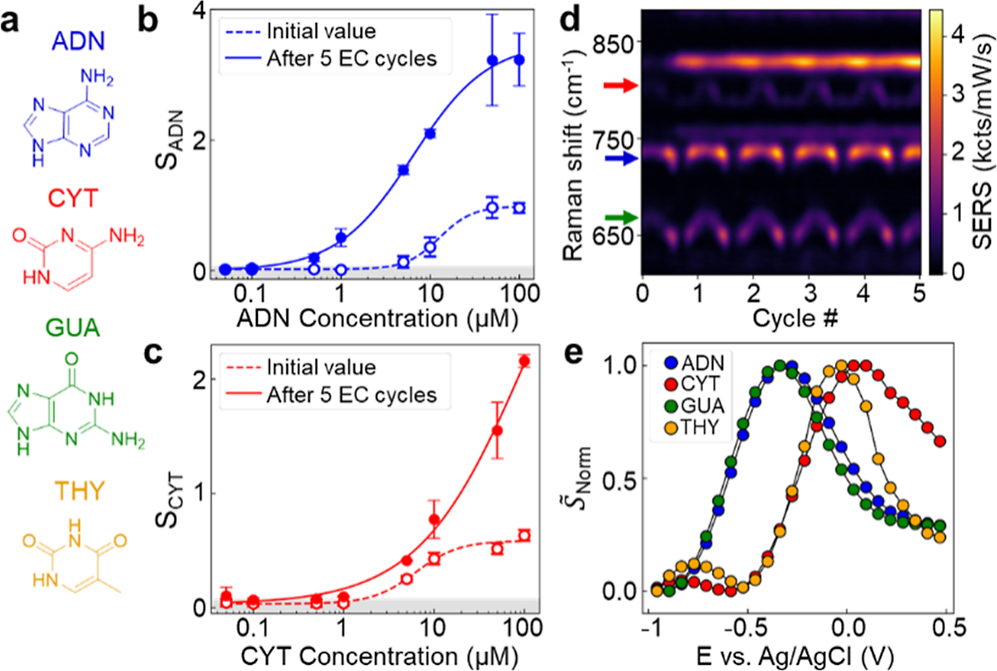
Multiplexed detection of DNA nucleobases. (**a**) Molecular structures of neutral adenine (ADN), cytosine (CYT), guanine (GUA), and thymine (THY). (**b,c**) Concentration series for detection of (b) ADN and (c) CYT. Peak areas (ADN 732 cm^–1^ and CYT 796 cm^–1^, normalized by CB[5]) are extracted before (open markers) and after (filled markers) 5 EC cycles (as shown in Figure 1). Error bars from standard deviation of 3 measurements, lines are Langmuir–Hill model fits, compared to noise level (gray). (**d**) Time-series SERS spectra while cycling MLagg in 5 μM ADN, 50 μM CYT, 50 μM GUA, and 50 μM THY in 500 mM KPB (pH 7.0). GUA, ADN, and CYT peaks at 660, 732, and 796 cm^–1^, respectively. THY peak at 1340 cm^–1^ in Figures S25–S27. (**e**) Final cycle intensities vs potential (vs Ag/AgCl) for each nucleobase.

A further feature of cyclic EC-SERS is the collapse in signal at more negative potentials (<−0.8 V for ADN and <−0.6 V for CYT), observed across all nucleobase vibrational modes but not in CB[5] peaks. Notably, the potential at which the signal loss occurs varies with the analyte, implying that it does not arise from electrode-driven processes such as hydrogen evolution or changes in interfacial ion composition (though H_2_ evolution may produce additional effects). Nor does it correlate with molecular p*K*_a_ values, ruling out deprotonation as the primary mechanism. Instead, we find that the drop-off potential closely tracks the rise potential: molecules that reorient at more positive potentials also show signal loss at more positive potentials (Figures 2 and 4e). Similarly, at higher analyte concentrations, where increased surface packing reduces flat adsorption energies^45^ and shifts the rise potential to more positive values, the drop-off potential also shifts similarly (Figure S19).

We thus suggest that the transition to a vertical orientation not only enhances the SERS signal but also weakens the molecular binding to the surface, making it susceptible to desorption. This behavior is consistent with electrochemical studies by Lipkowski and co-workers, who showed that aromatic adsorbates such as pyrazine undergo potential-dependent reorientation at gold electrodes, transitioning from flat to vertical configurations before desorbing at more negative potentials.^18,19^ QM/MM simulations provide molecular-level insight into this process: as molecules reorient vertically, K^+^ ions actively coordinate with the analytes, stabilizing the vertical configuration (Figure 3f). However, the transition to a vertical orientation also weakens the van der Waals contact with the surface. At sufficiently negative potentials, repulsion between the molecular dipole and the negatively charged electrode overcomes the ion-mediated stabilization, driving desorption. Support for this potential-driven desorption model comes from cyclic EC-SERS experiments where the scan rate is varied. At fast scan rates (>100 mV/s) the drop-off in SERS signal is minimal, suggesting there is insufficient time for molecules to desorb and diffuse away from each nanogap (Figure S20). In contrast, holding the potential longer at negative values gives a pronounced and often irreversible loss of signal, consistent with molecular desorption and diffusion out of the nanogap which depends on the dwell time and flow conditions (Figures S21 and S22). The hysteresis consistently observed in both signal intensity and peak position during cycling (Figure 2) can be attributed to the time required for desorbed molecules to diffuse back into the nanogaps, readsorbing as the potential becomes less negative. This hysteresis diminishes at slower scan rates, where longer time scales allow for molecular diffusion and readsorption (Figures S9 and S10).

The final observation to account for is the sustained increase in signal over multiple cycles, which cannot simply be explained by diffusion (Figures S11 and S22). We propose this enhancement arises from molecular reordering at the Au surface (Figure 3g). Initially at OCP, analytes are randomly adsorbed flat. Applying negative potential reorients the molecules vertically, lifting them to enable more efficient packing and creating space for additional analyte adsorption. This restructuring not only increases the number of molecules in the nanogap but may also promote ordered intermolecular interactions, such as π–π stacking, further amplifying SERS signals. In contrast, when the potential is held constant, reorientation occurs, but this dynamic reordering mechanism is inactive, limiting signal enhancements (Figure S11). This model successfully predicts that fully bound CB[5] molecules show no significant SERS increase (Figure S6). We thus propose that potential cycling not only modulates analyte orientation, giving rise to the observed oscillations in peak intensity and position, but also actively drives molecular reorganization, enabling progressive signal enhancement across successive cycles. The dynamic reversal of the EDL thus accumulates analytes onto the surface.

For SERS to be effective for low-cost rapid healthcare sensing, it must be able to quantify very low concentrations of biomolecules and multiple analytes simultaneously. Cyclic EC-SERS improves both these capabilities. We first investigate the LoDs of nucleobases by performing cyclic voltammetry of solutions containing 0.05–100 μM ADN or CYT in 500 mM KPB while recording the SERS spectra of the MLagg. Before introducing an analyte, the MLagg is cleaned and scaffolded using the ReSERS procedure.^26^ After adding the analyte solution to the EC-SERS cell and letting equilibrate at OCP (Figure S22), it is cycled between +0.5 V and −1 V at 50 mV s^–1^ for 5 cycles. Three EC-SERS measurements are recorded for each solution, and the spectra are normalized using the CB[5] 828 cm^–1^ peak recorded post-ReSERS cleaning.

Cyclic EC-SERS consistently increases the magnitude of the characteristic nucleobase peaks and enables the detection of concentrations which have no visible analyte peaks at OCP. To determine the LoDs, the maximum CB[5] normalized peak areas (ADN 732 cm^–1^ and CYT 796 cm^–1^) after 5 cycles are extracted (Figure 4b,c). These are then fit to a Langmuir–Hill model (Supporting Information Section S2), and the LoD is determined from its intersection with the 3σ confidence band of the noise level. The LoD for ADN after cyclic EC-SERS is 147 nM compared to 3.7 μM before cycling the potential. For CYT, the LoD is 260 nM with cyclic EC-SERS and 1.7 μM without (Figure 4b,c).

Finally, we perform simultaneous detection of all four DNA nucleobases: ADN, CYT, guanine (GUA), and thymine (THY) (Figure 4a). Analyte solutions containing 50 μM of CYT, GUA, THY, and 5 μM ADN in 500 mM KPB are cycled as above. A lower ADN concentration is used as its large Raman cross section dominates EC-SERS spectra for equal analyte concentrations (Figure S25).^27,28^ We observe and distinguish between the peaks of all four nucleobases (Figure 4d), with each exhibiting unique peak oscillations (Figures 4e and S24–S26). The purines (ADN and GUA) display similar behavior (*V*_max_≃-0.4 V) as do the pyrimidines (CYT and THY) with *V*_max_≃0 V. These groupings support our proposed mechanism that the reorientation potential of each analyte is governed by a balance between flat-surface adsorption energy and the energetic stabilization gained through vertical alignment with the interfacial electric field. Purines, with their extended π-conjugated aromatic systems, exhibit stronger π–metal interactions when adsorbed flat on the gold surface, resulting in a higher energetic barrier to reorientation. In contrast, pyrimidines possess smaller π-systems, have weaker surface interactions, and reorient more readily at less negative potentials.

Our mechanistic understanding provides a framework for interpreting analyte-specific EC-SERS responses and can be extended to a much broader class of molecules. By analyzing the spectro-electrochemical signatures of different analytes using advanced data-driven analysis (including machine learning), this platform offers a route to develop both a deeper understanding of ionic double-layer interactions with analytes, as well as improved spectral discrimination between analytes.

## Conclusions

In conclusion, we demonstrate that low-cost recleanable SERS substrates enable study of the unique EC-SERS behavior of neutral molecules at metal surfaces. Simulations reveal the potential-induced reorientation of molecules and their dynamic interaction with the locally structured EDL. This approach addresses key challenges in SERS sensing and enables multiplexed detection in complex fluids. We find that cyclic EC-SERS significantly reduces the LoDs of nucleobases (so far <100 nM) and enables their simultaneous multiplexed detection. How the double layer at electrodes wraps around molecules, and how this changes as their ion layers invert with potential, is crucial across a wide range of fields.

## Methods

### Materials

All chemicals were used as received. Citrate-stabilized 80 nm AuNPs (optical density 1.0 at 555 nm) were purchased from BBI Solutions. Analytical-grade chloroform (≥99.8%) was obtained from Merck. K_2_HPO_4_ (≥98%) and KH_2_PO_4_ (≥98%) were from Alfa Aesar. Cucurbit[5]­uril hydrate (≈20% water), adenine (ADN, ≥99%), cytosine (CYT, ≥99%), guanine (GUA, ≥99%), and thymine (THY, ≥99%) were obtained from Sigma-Aldrich. Polydimethylsiloxane (PDMS) was prepared using a SYLGARD 184 kit from DOWSIL (Dow Silicones). Fluorine-doped tin oxide (FTO)-coated glass slides (TEC 10) were purchased from Ossila Ltd. and were cleaned and cut to 10 × 15 mm^2^ slides prior to use. All aqueous solutions were prepared using deionized (DI) water (>18.2 MΩ cm^–1^) from a Purelab Ultra Scientific water purification system.

### Monolayer Aggregate Preparation

MLagg substrates were prepared by mixing 500 μL citrate-stabilized 80 nm AuNPs with equal volume chloroform and initiating aggregation with the addition of 20 μL of 2 mM CB[5].^24,25^ Aggregation was facilitated with 1 min of vigorous shaking, after which the aggregates settled at the liquid–liquid interface. Excess ligands and salts were removed by replacing the aqueous supernatant with fresh DI water. This washing step was repeated three times. The aggregates were then concentrated by carefully decreasing the volume of the aqueous phase to ∼20 μL. The AuNP aggregate was then transferred via a pipette to a cleaned FTO-coated glass slide and allowed to air-dry.

### SERS

SERS measurements were recorded on a custom-built Raman setup with an Andor Newton 970 EMCCD camera coupled to a Shamrock 168 spectrometer and a Matchbox 785 nm diode laser (Figure S2). Excitation and collection were performed through an Olympus LUMPlanFl/IR × 40 W NA 0.80 water-immersion objective (in inverted configuration) at 1 s integration times with 1 mW laser power.^26^

### EC-SERS

An EC-SERS cell was fabricated from PDMS to accommodate a three-electrode electrochemical system: a Pt wire (Sigma-Aldrich) counter electrode, a leakless Ag/AgCl/KCl (LF-1-45 from Innovative Instruments Ltd.) reference electrode, and an MLagg SERS substrate on FTO-coated glass as the working electrode (Figures S1 and S2).^26^ The electrolyte compartment was defined by using a 6 mm diameter biopsy punch. Using custom 3D-printed stage holders, the EC-SERS cell was sealed and mounted onto the stage of an inverted Raman setup, with SERS probed from below the cell. Electrochemical measurements were conducted using a portable potentiostat (Rodeostat) from IO Rodeo. All potentials were referenced to a Ag/AgCl reference electrode.

### Cleaning and Regeneration of MLaggs

To clean and regenerate the MLagg,^26^ 50 mM potassium phosphate buffer (pH 7.0) was pipetted into the EC-SERS cell, and a potential of +1.5 V vs Ag/AgCl was applied for 60 s. After cleaning, the original buffer solution was removed, and 1 mM CB[5] in 50 mM potassium phosphate buffer (pH 7.0) was pipetted to the cell. A potential of −0.80 V vs Ag/AgCl was then applied for 15 s. The EC-SERS cell was then washed out with buffer before performing the next measurement. If traces of previously detected analyte were evident from the SERS spectrum, another round of cleaning/regeneration was conducted.

### Scanning Electron Microscopy Measurements

Scanning electron microscopy (SEM) imaging of MLaggs deposited on FTO-coated glass was conducted using a FEI Philips Dualbeam Quanta 3D SEM (dwell 3–10 μs, HV 2 kV, current 50 pA, and ≈2.0 mm WD).

### Data Analysis

SERS spectra were background-corrected using asymmetric least-squares (ALS) baseline correction. Analyte peak areas and center points were determined by defining a spectral region and then fitting Gaussian curves to the peaks of interest. CB[5]-normalized peak areas were calculated using the 828 cm^–1^ CB[5] peak from the clean rescaffolded MLagg spectra recorded before adding the analyte of interest.

### Mean-Field QM/MM Simulations

Au­(111) electrode–KPB electrolyte interfaces were simulated using density functional theory in classical explicit solvents 2 (DFT-CES2), which is the mean-field QM/MM simulation method.^39^ DFT-CES2 demonstrates chemical accuracy in describing interfacial interactions. Moreover, DFT-CES, the previous version of DFT-CES2,^46,47^ has been used to accurately describe various electrochemical interface systems such as the electric double layer, electrocatalysts, etc.^46,47^ Details of the method can be found in Supporting Information Section S1.

The Au(111) electrode was modeled at the QM level using DFT. Here, Au(111) consists of a four-layer slab with 32 atoms with a dimension of 10.18 × 5.88 Å^2^. The projector-augmented-wave (PAW) method was applied with a kinetic energy cutoff of 50 Ry.^48^ The Perdew–Burke–Ernzerhof (PBE) exchange–correlation functional was used.^49^ Gaussian smearing was used with a value of 0.0147 Ry. A (5 × 9 × 1) Γ-centered k-point grid was used to sample the Brillouin zone. A dipole correction along the *z*-direction was applied.

Classical molecular dynamics (MD) simulations were employed to describe the electrolyte phase. The dimension of the MD simulation cell size was set to 40.7 × 47.0 × 65.0 Å^3^, which corresponds to the (4 × 8) supercell of the QM simulation cell. Within these 2200 water molecules, an additional 3 K^+^, 1 HPO_4_^2–^, and 1 H_2_PO_4_^–^ were included as appropriate for the 50 mM KPB at pH 7.0. We further added 1 adenine or cytosine molecule in the MD simulation cell. The simulations do not include the CB[5] scaffold molecules used experimentally to define the nanogaps. This simplification reduces computational cost and isolates the fundamental analyte–ion–surface interactions. The validity of this approximation is supported by the minimal spectral response of CB[5] to potential cycling observed experimentally (Figures S5 and S6). Additionally, the simulations model a flat Au(111) surface rather than the nanogap geometry present experimentally. The 80 nm nanoparticles present extended facets that are large (∼20 nm) compared to the analyte molecules; therefore, molecules adsorbing on these facets experience a locally flat environment.

The TIP4P-EW water model is used to describe the water–water interaction.^50^ Ion–ion and ion–water interactions are described using the parameters from previous studies.^51,52^ Dreiding force field parameters are used to describe the adenine and cytosine molecules.^53^ Additional ions are added to compensate for the non-neutral charge of the Au electrode. A Nosé–Hoover thermostat was used to maintain the temperature at 300 K.^54,55^ To account for long-range electrostatic interactions, the modified particle–particle particle-mesh (PPPM) method was applied with periodic boundary conditions along the *x*- and *y*-directions.^56^

## Supplementary Material



## Data Availability

All data needed to evaluate the conclusions in the paper are present in the paper and/or the Supporting Information.

## Notes

DFT-CES2 simulation methodology; scanning electron micrographs of the SERS substrates; schematic of the electrochemical SERS setup; additional cyclic voltammetry and EC-SERS measurements; analysis of scan-rate, electrolyte, buffer concentration, and potential-dependent effects; molecular dynamics simulation results and nucleobase orientation calculations; Langmuir–Hill modeling and fit parameters; and additional EC-SERS data for adenine, cytosine, guanine, thymine, and multiplexed nucleobase detection (PDF)
