# Supplementary material for: How the Electrochemical Double Layer Manipulates Molecule–Metal Interactions
Source: ACS Nano. 2026 Jun 18;20(25):18284–92. doi: 10.1021/acsnano.6c03605 (PMC13325844; doi:10.1021/acsnano.6c03605)
Supplement: Supplementary file 1 [file nn6c03605_si_001.pdf]

How the electrochemical double layer manipulates molecule-metal interactions

Tabitha Jones[1], Minho M. Kim[2], Sarah May Sibug-Torres[1], Elle Wyatt[1], Nicolas Spiesshofer[1], James W. Beattie[1], Jonathan Bar-David[1], Rakesh Arul[1], Bart de Nijs[3], Hyungjun Kim[2*], Jeremy J. Baumberg[1*]

[1] NanoPhotonics Centre, Cavendish Laboratory, Department of Physics, JJ Thompson Avenue, University of Cambridge, Cambridge, CB3 0HE, United Kingdom
[2]Department of Chemistry, Korea Advanced Institute of Science and Technology, Daejeon, 34141, Republic of Korea
[3]Physics for Sustainable chemistry group, Cavendish Laboratory, Department of Physics, JJ Thompson Avenue, University of Cambridge, Cambridge, CB3 0HE, United Kingdom

* Corresponding authors: jjb12@cam.ac.uk, linus16@kaist.ac.kr

Supplementary material

S1: Brief summary of DFT-CES2 simulation method……………………………………………………………2
Fig. S1: Scanning electron micrographs (SEM) of the MLagg-CB[5]………………………………………4
Fig. S2: Schematic diagram of the electrochemical SERS set-up…………………………………………5
Fig. S3: Cyclic voltammetry in different electrolytes……………………………………………………………5
Fig. S4: Cyclic electrochemical SERS of adenine for 7 cycles……………………………………………6
Fig. S5: Cyclic electrochemical SERS of potassium phosphate buffer ……………………………6
Fig. S6: EC-SERS response of CB[5]……………………………………………………………………………………7
Fig. S7: Oxidation of gold during EC cycling…………………………………………………………………7
Fig. S8: Cyclic electrochemical SERS of adenine at different scan rates………………………8
Fig. S9: Comparison of ADN peak signals during 5 mV/s scan……………………………………8
Fig. S10: Comparison of ADN peak positions during 5 mV/s scan………………………………9
Fig. S11: Comparison of different EC conditions……………………………………………………………9
Fig. S12: Cyclic electrochemical SERS of cytosine…………………………………………………10
Fig. S13: EC-SERS response of cytosine…………………………………………………………………………11
Fig. S14: Relationship between $V_{\max}$ and pKa for different nucleobases……………………………12
Fig. S15: Effect of laser illumination during potential cycling……………………………………12
Fig. S16: Ion distributions from molecular dynamics simulations………………………………13

Fig. S17: Effect of buffer concentration on cyclic EC-SERS response…………………………….…..14
Fig. S18: Simulated orientation of nucleobases on Au (111) at different applied potentials..…..14
Fig. S19: Comparison of ADN peak signals for different concentrations…………………………….15
Fig. S20: Comparison of ADN peak signals during EC scans of different rates……………………15
Fig. S21: Effect of applying constant negative potential………………………………………………….16
Fig. S22: Adenine desorption with application of constant negative potential……………………16
Fig. S23: Effect of waiting at OCP before performing cyclic EC-SERS………………………………..17
S2. Langmuir-Hill model……………………………………………………………………………………………17
Table S1: Langmuir-Hill fit coefficients for adenine and cytosine……………………..……………..17
Fig. S24: Cyclic electrochemical SERS of guanine……………………………..…………………….18
Fig. S25: EC-SERS response of guanine…………………………..……………..……………………18
Fig. S26: Cyclic electrochemical SERS of thymine……………………..………………………………19
Fig. S27: EC-SERS response of thymine……………………..………………………………..…………20
Fig. S28: Cyclic electrochemical SERS of multiple DNA nucleobases……………………..…………21

S1: Brief summary of DFT-CES2 simulation method

Density functional theory in classical explicit solvents 2 (DFT-CES2) is a mean-field QM/MM simulation method which accurately models the electrostatic, Pauli repulsion, and dispersion interactions between QM and MM regions.[1] The total Hamiltonian ($H_{QM/MM}$) is described as the sum of the QM Hamiltonian (H_{QM}), MM Hamiltonian (H_{MM}), and interaction energy ($E_{\mathrm{Int}}^{\mathrm{QM/MM}}$)

$$H_{\mathrm{QM/MM}} = H_{\mathrm{QM}} + H_{\mathrm{MM}} + E_{\mathrm{Int}}^{\mathrm{QM/MM}} \quad (1)$$

$$H_{\mathrm{QM}} = \sum_{a \in A} \frac{\boldsymbol{p}_a^2}{2m_a} + E^A\left[\rho^A; \{\boldsymbol{r}_a\}\right] \quad (2)$$

$$H_{\mathrm{MM}} = \sum_{b \in B} \frac{\boldsymbol{p}_b^2}{2m_b} + E^{FF}\left(\{\boldsymbol{r}_a\}\right) \quad (3)$$

$$E_{\mathrm{Int}}^{\mathrm{QM/MM}} = E_{\mathrm{disp}} + E_{\mathrm{Coul}} + E_{\mathrm{Pauli}} \quad (4)$$

Here, A is the QM subsystem and B is the MM subsystem. $\boldsymbol{r}$ and $\boldsymbol{p}$ are the position and momentum vectors, respectively. m is the mass and ρ^A is the electron density of subsystem A. E^A and E^{FF} are the Kohn-Sham DFT energy and the force-field energy, respectively.

Dispersion energy, E_{disp}, is described using Grimme's DFT-D3 scheme along with the Becke-Johnson (BJ)-damping function:[2,3]

$$E_{\text{disp}} = -s \sum_{a \in A, b \in B} \frac{C_{6,ab}}{\boldsymbol{r}_{ab}^{6} + f(R_{ab})^{6}} (5)$$

where $f(R_{ab}) = a_0 R_{ab}^{0} + a_1$, and $\{\boldsymbol{r}_a\}$ represents the nuclei position of the subsystem A. The detailed parameter determination can be found in the original paper.[1]

The Coulomb interaction energy, E_{Coul} is defined as:

$$E_{\text{Coul}} = \int \mathrm{d}\boldsymbol{r}^{3} v_{\text{Coul}}(\boldsymbol{r}) \left\{\rho_{\text{nuc}}^{A}(\boldsymbol{r}) - \rho^{A}(\boldsymbol{r})\right\} (6)$$

where ρ_{nuc}^{A} is the nuclear density of subsystems A, and v_{Coul} is the electrostatic potential generated by subsystem B. Here, we neglect the induction energy due to its small contribution compared with the Coulomb interaction.

Lastly, the Pauli repulsion, E_{Pauli}, is defined as the electron density overlap between subsystems A and B:

$$E_{\text{Pauli}} = \int \mathrm{d}^{3}\boldsymbol{r} v_{Pauli} \tilde{\rho}^{A}(\boldsymbol{r}) (7)$$

$$v_{Pauli}(\boldsymbol{r}_b;\sigma_b) = \sum_{b \in B} a_b g(\boldsymbol{r} - \boldsymbol{r}_b;\sigma_b) (8)$$

where $\tilde{\rho}^{A}$ is the electron density of unperturbed (nonorthogonalized) orbitals, and a_b is the repulsion parameter. Here g is the Gaussian function, and σ is its size, with the electron density of subsystem B being described with a Gaussian shape.

The DFT-CES2 (or DFT-CES) method employs a mean-field approximation to avoid the computationally intensive dynamic simulation of the full QM system. The electrostatic and Pauli potential of the QM system is applied as an external potential to the MM system, while the ensemble-averaged electrostatic and Pauli potentials sampled during the MD simulation are applied as an external potential to the DFT optimization. This quantum mechanics-based embedding scheme is distinct from DFT-CES, which only includes the electrostatic embedding scheme. A real-space grid is employed as the communication channel between the QM/MM. DFT optimizations and MD simulations are performed iteratively until the energy converges.

DFT-CES2 is implemented by combining Quantum Espresso, as the engine for DFT optimization, and the Large-scale Atomic/Molecular Massively Parallel Simulator (LAMMPS),[4,5] as the engine for MD simulations. More details can be found in previous papers.[1,6]

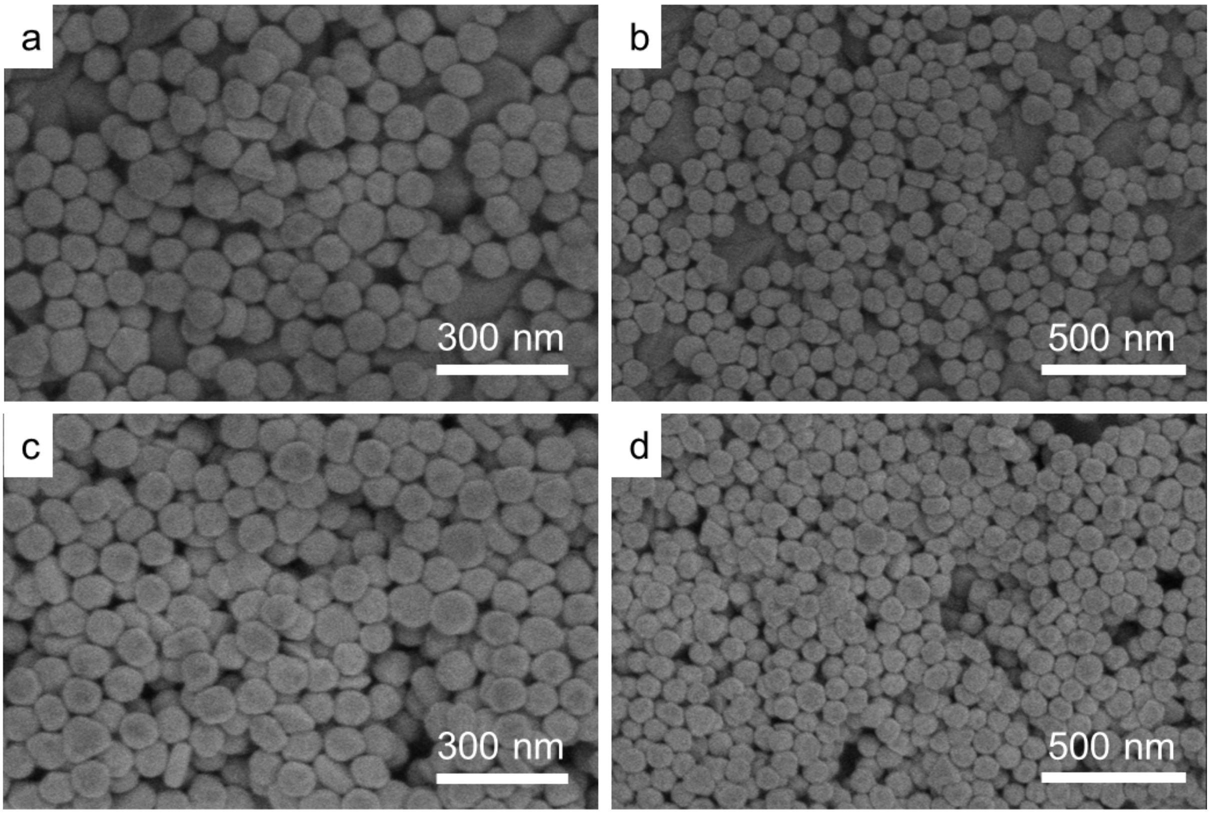

Fig. S1: **Scanning electron micrographs (SEM) of the MLagg-CB[5] substrate** after (a, b) initial cleaning and regeneration, and (c, d) following electrochemical cycling between +0.5 V and –1 V in 10 μM adenine and 50 mM potassium phosphate buffer (pH 7.0) at 50 mV s^{-1} for five cycles.

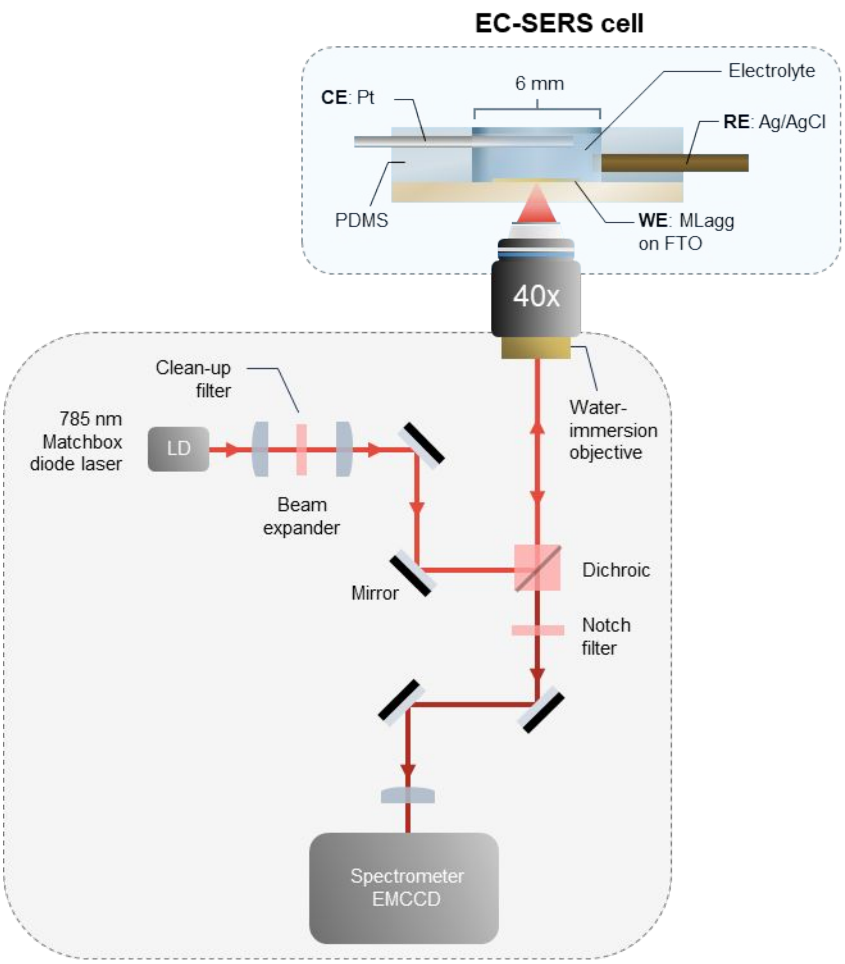

Fig. S2: **Schematic diagram of electrochemical SERS (EC-SERS) set-up** including cross-section of the spectro-electrochemical cell. CE = counter electrode, RE = reference electrode, and WE = working electrode.

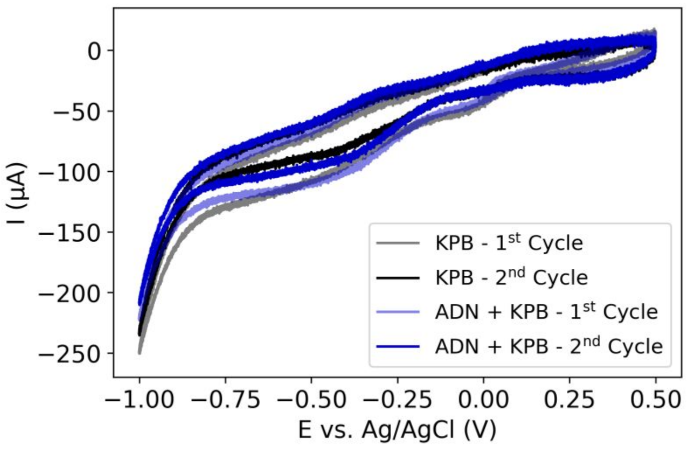

Fig. S3: **Cyclic voltammetry in different electrolytes.** Overlaid cyclic voltammograms of MLaggs in 50 mM potassium phosphate buffer (pH 7.0) (black) and 10 µM adenine (ADN) and 50 mM potassium phosphate buffer (pH 7.0) (blue). The applied potential is cycled from 0 V (*vs* Ag/AgCl) to +0.5 V, down to -1 V and then back to 0 V at a scan rate of 50 mV s^{-1}. The first two cycles are plotted here.

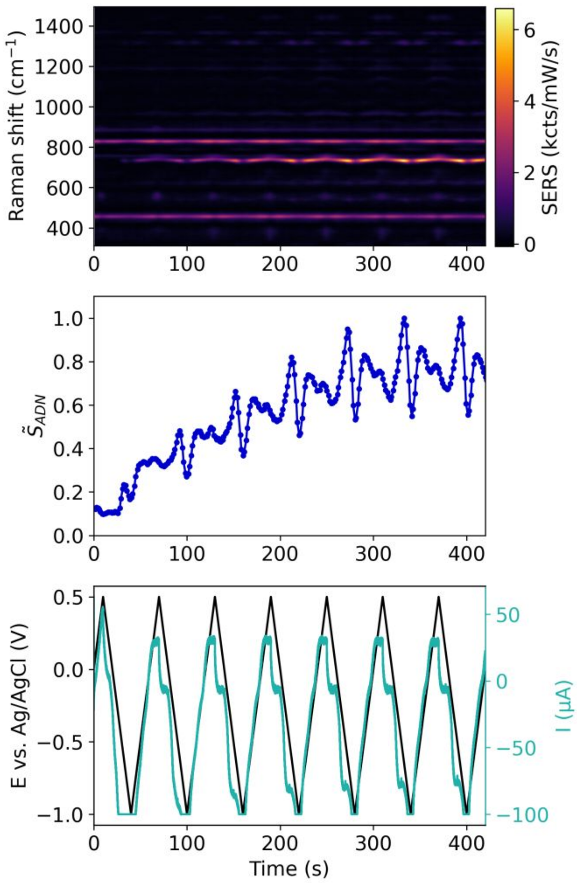

Fig. S4: Cyclic electrochemical SERS of adenine for 7 cycles: Time-series SERS spectra (1 s integration time, 785 nm 1 mW laser) of an MLagg cycled between +0.5 V and -1 V in 10 μM adenine (ADN) and 50 mM potassium phosphate buffer (pH 7.0) for seven cycles. Normalised peak area for ADN peak at ~732 cm^{-1} (blue), applied potential (black), and current (cyan) plotted vs time.

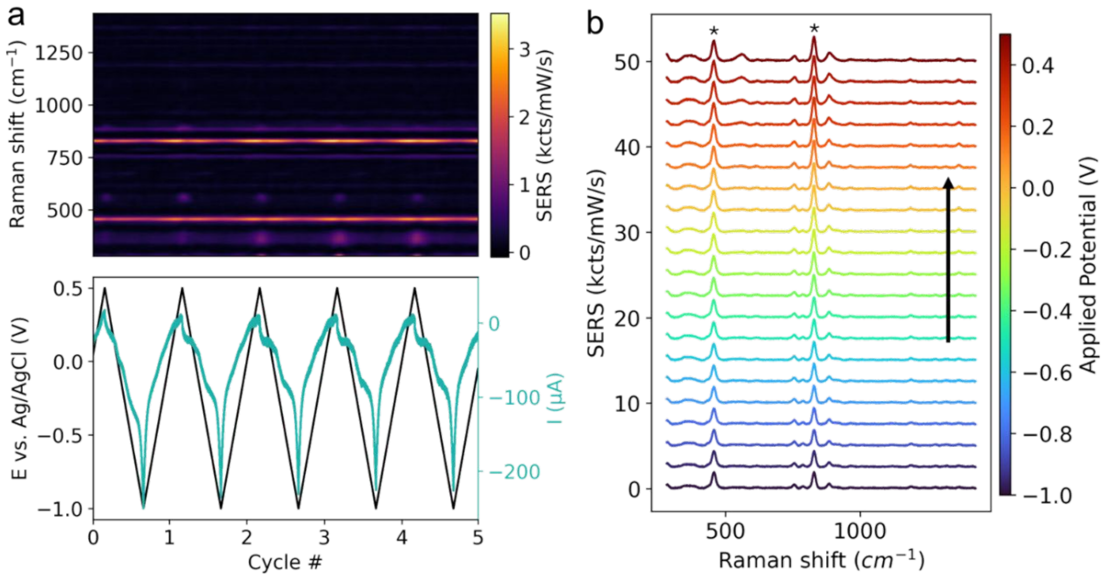

Fig. S5: Cyclic electrochemical SERS of potassium phosphate buffer using an MLagg substrate. (a) Time-series SERS spectra (1 s integration time, 785 nm 1 mW laser) of the MLagg cycled between +0.5 V and -1 V in 50 mM potassium phosphate buffer (pH 7.0) at 50 mV s^{-1} for 5 cycles. (b) Spectra taken from the first positive cycle from -1 V to +0.5 V. Characteristic CB[5] peaks are indicated by *.

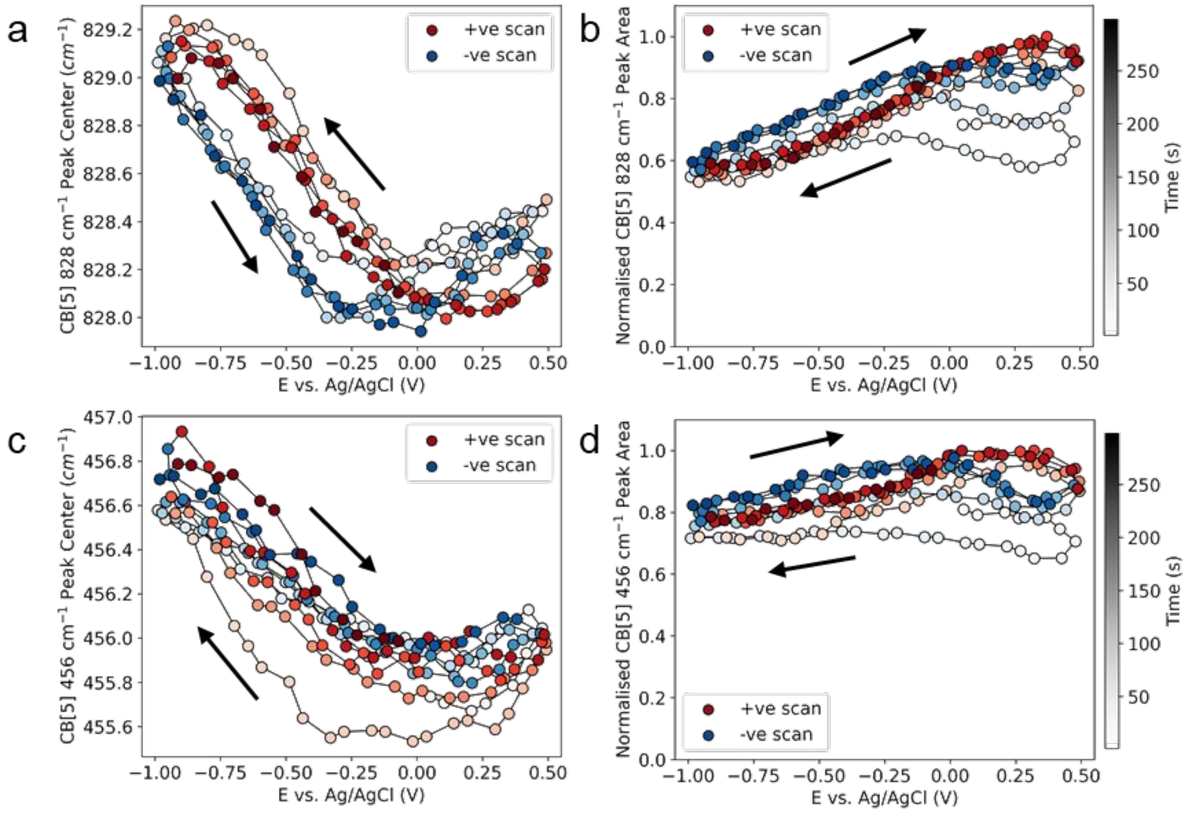

Fig. S6: EC-SERS response of CB[5]. (a, c) Peak frequency *vs* applied potential (*vs* Ag/AgCl) for the peaks at (a) 828 cm^{-1} and (c) 456 cm^{-1}, which are attributed to CB[5] in the scaffolded MLagg, conditions and cycling as Fig. S4. (b,d) Normalised SERS peak area *vs* applied potential for the same peaks as (a,c). Intensity of marker colour denotes the time elapsed, arrows indicate cycle direction.

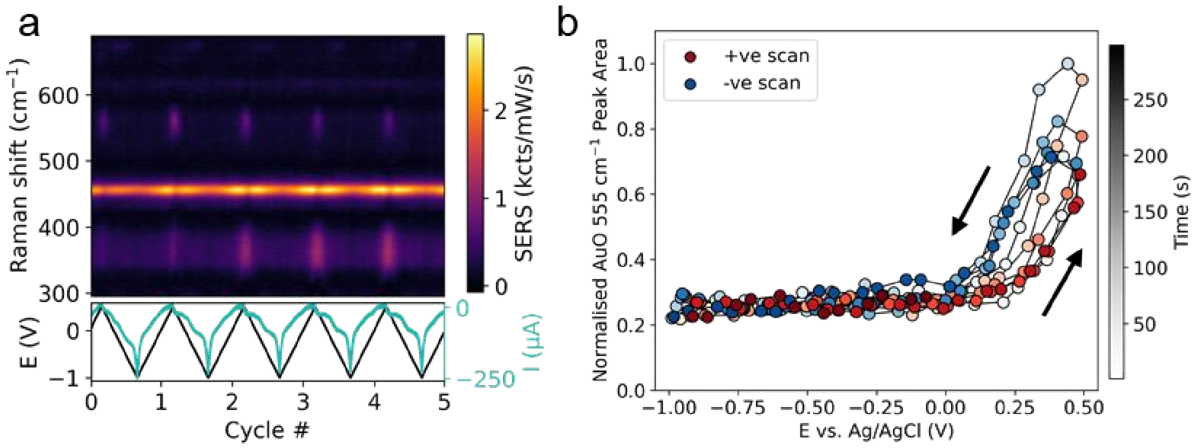

Fig. S7: Oxidation of gold during EC cycling. (a) Time-series SERS spectra (zoomed-in) of a 50 mM potassium phosphate buffer solution (pH 7.0), conditions and cycling as Fig. S4. (b) Normalised SERS peak area *vs* applied potential (*vs* Ag/AgCl) for the gold oxide peak at 555 cm^{-1}. Marker hue denotes the time elapsed, arrows indicate cycle direction.

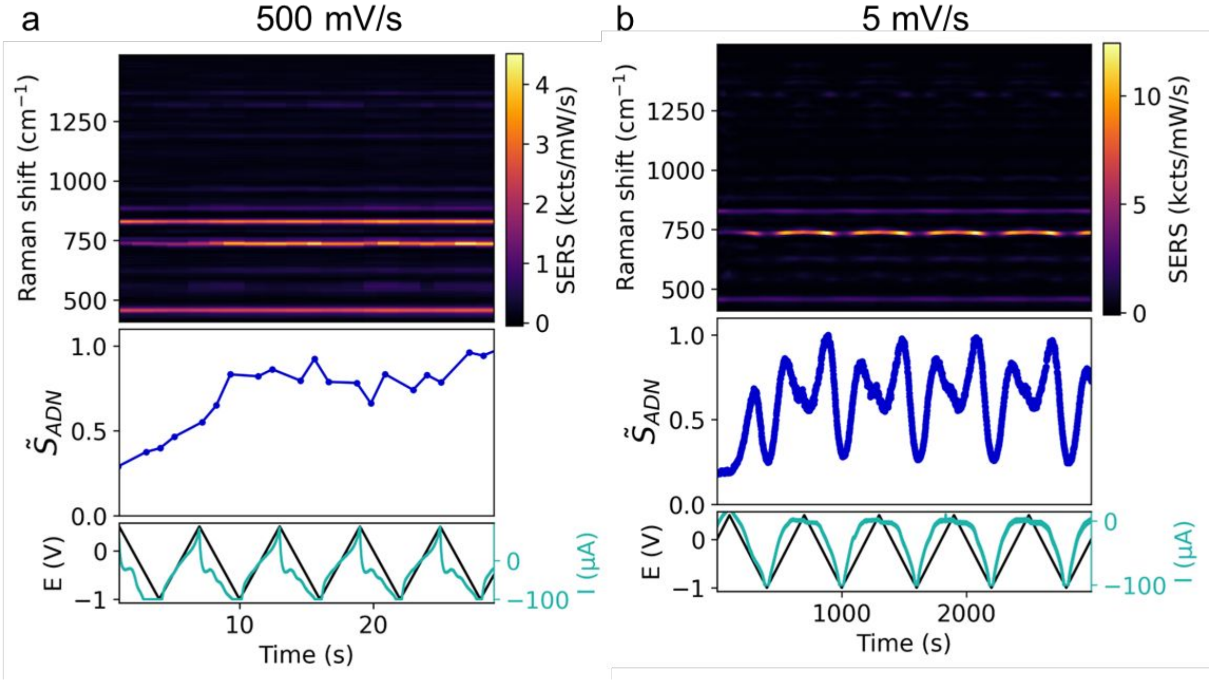

Fig. S8: Cyclic electrochemical SERS of adenine at different scan rates. Time-series SERS spectra (1 s integration time, 785 nm 1 mW laser) of the MLagg cycled between +0.5 V and -1 V in 10 µM adenine (ADN) and 50 mM potassium phosphate buffer (pH 7.0) at (a) 500 mV s^{-1} and at (b) 5 mV s^{-1} for 5 cycles. Normalised peak area for ADN peak at ~732 cm^{-1} (blue), applied potential (black), and current (cyan) plotted vs time.

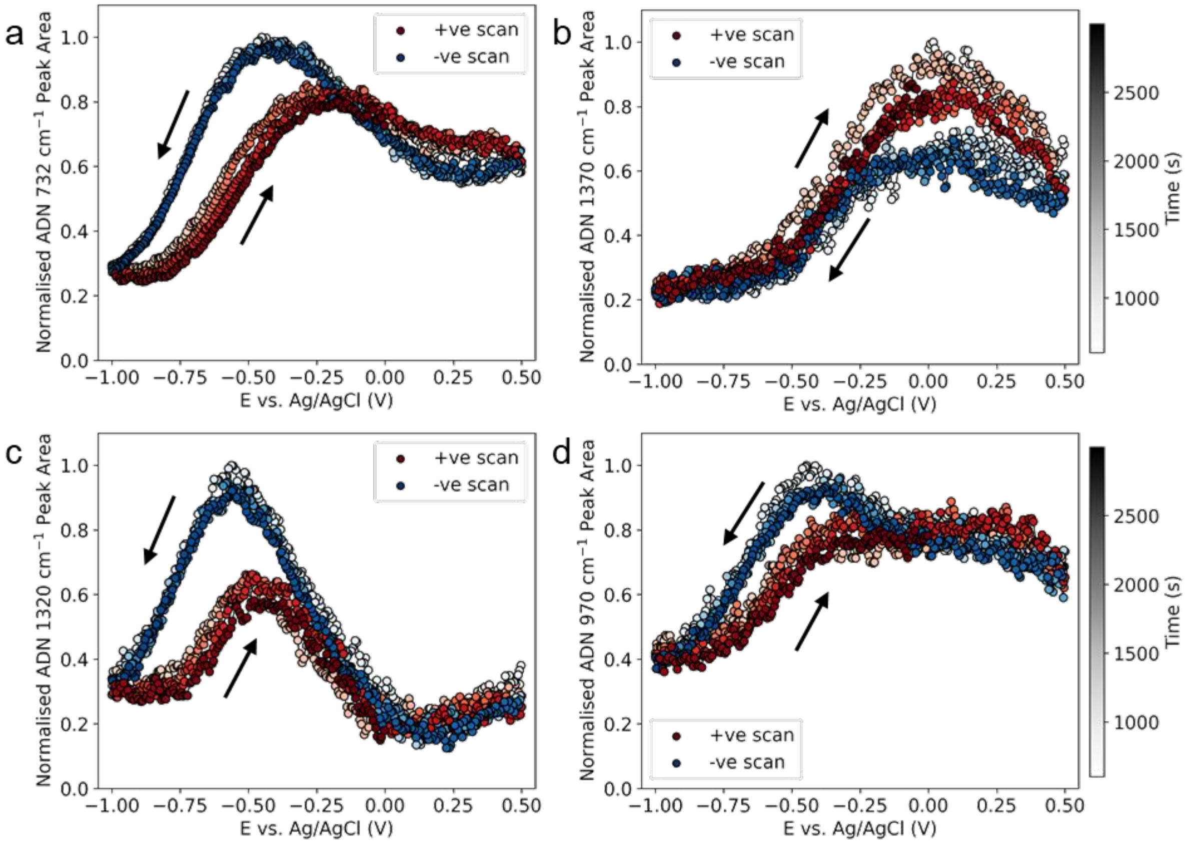

Fig. S9: Comparison of ADN peak signals during 5 mV/s scan. Normalised SERS peak area *vs* applied potential (*vs* Ag/AgCl) for the peaks at (a) 732 cm^{-1} (ring-breathing), (b) 1370 cm^{-1} (N-C-H in-plane bending), (c) 1320 cm^{-1} (C-N stretch), (d) 970 cm^{-1} (5-ring deformation. Conditions and cycling as Fig. S7b, marker hue denotes the time elapsed.

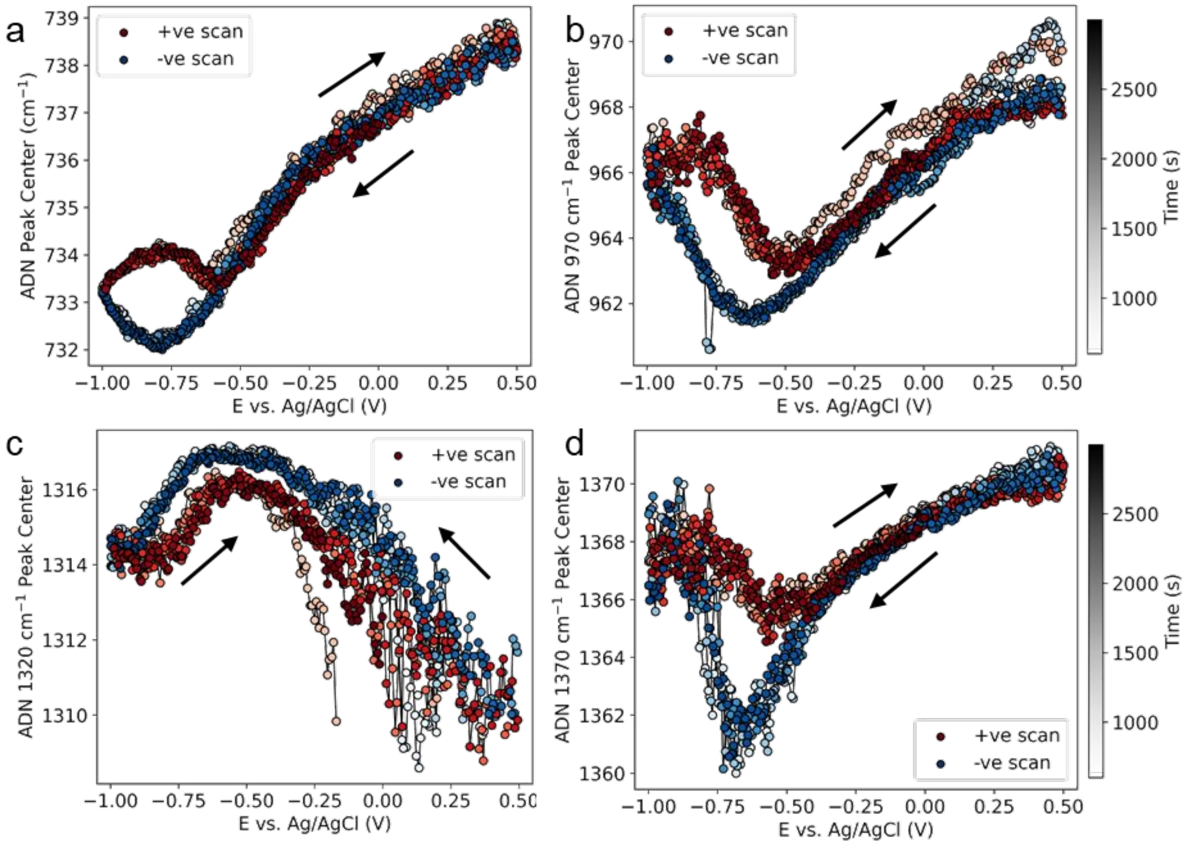

Fig. S10: Comparison of ADN peak positions during 5 mV/s scan. SERS peak center *vs* applied potential (*vs* Ag/AgCl) for the peaks at (a) 732 cm^{-1} (ring-breathing), (b) 1370 cm^{-1} (N-C-H in-plane bending), (c) 1320 cm^{-1} (C-N stretch), (d) 970 cm^{-1} (5-ring deformation). Conditions and cycling as Fig. S7b, marker hue denotes the time elapsed.

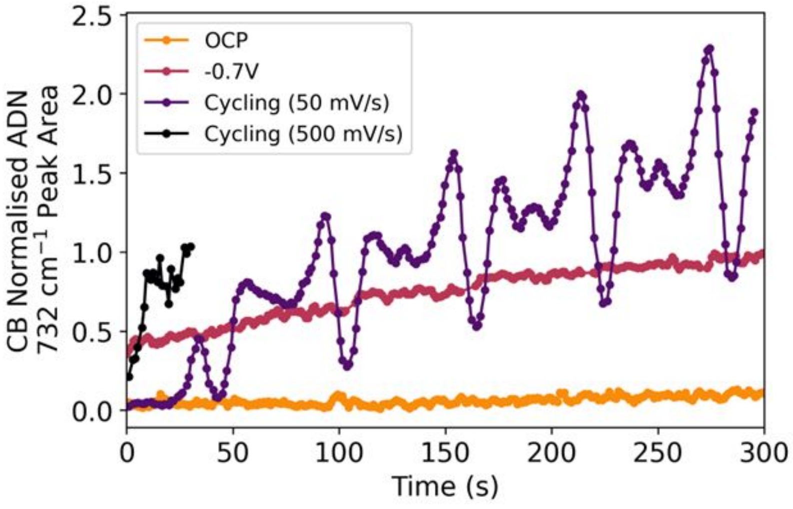

Fig. S11: Comparison of different EC conditions. ADN peak area (~732 cm^{-1}, normalised by CB[5]) plotted against time for different EC conditions: waiting at open circuit potential (OCP) (orange),

applying a constant potential of -0.7V (pink), cycling between +0.5 V and -1 V at 50 mV/s (purple) and 500 mV/s (black). The electrolyte was 10 μM ADN and 50 mM potassium phosphate buffer (pH 7.0).

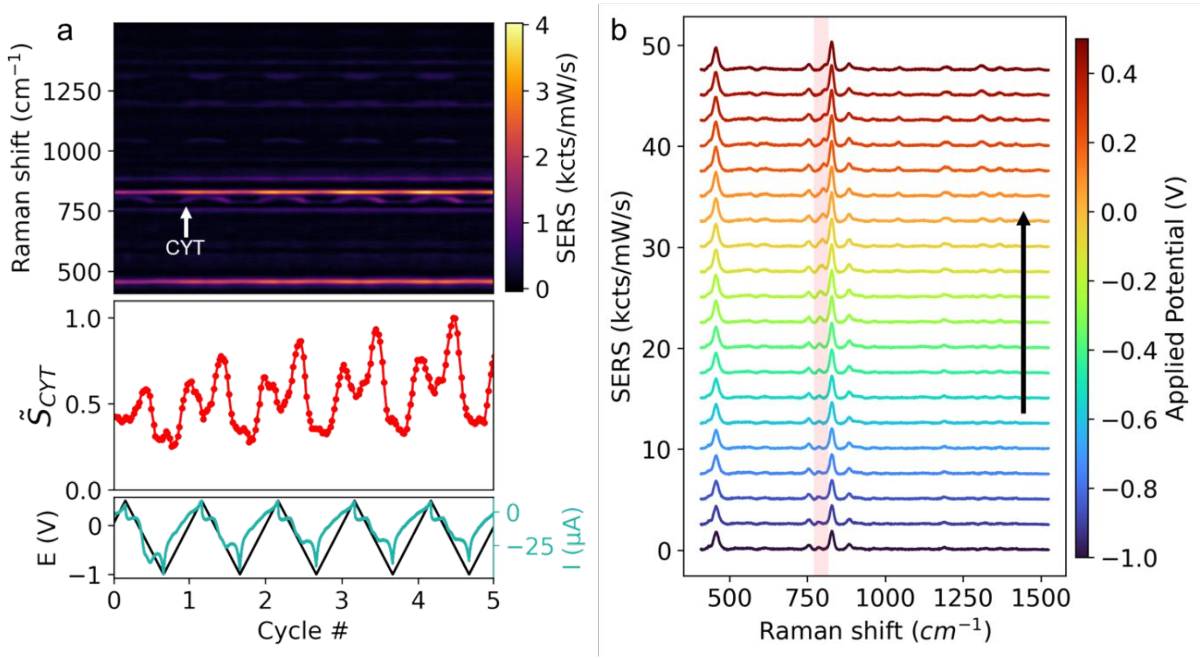

Fig. S12: Cyclic electrochemical SERS of cytosine. (a) Time-series SERS spectra (1 s integration time, 785 nm 1 mW laser) of the MLagg cycled between +0.5 V and -1 V in 10 μM cytosine (CYT) and 50 mM potassium phosphate buffer (pH 7.0) at 50 mV s^{-1} for 5 cycles. Normalised peak area for CYT peak at 796 cm^{-1} (red), applied potential (black), and current (cyan) plotted vs time. (b) Spectra taken from the first positive cycle from -1 V to +0.5 V. The characteristic CYT peak is shaded in pink.

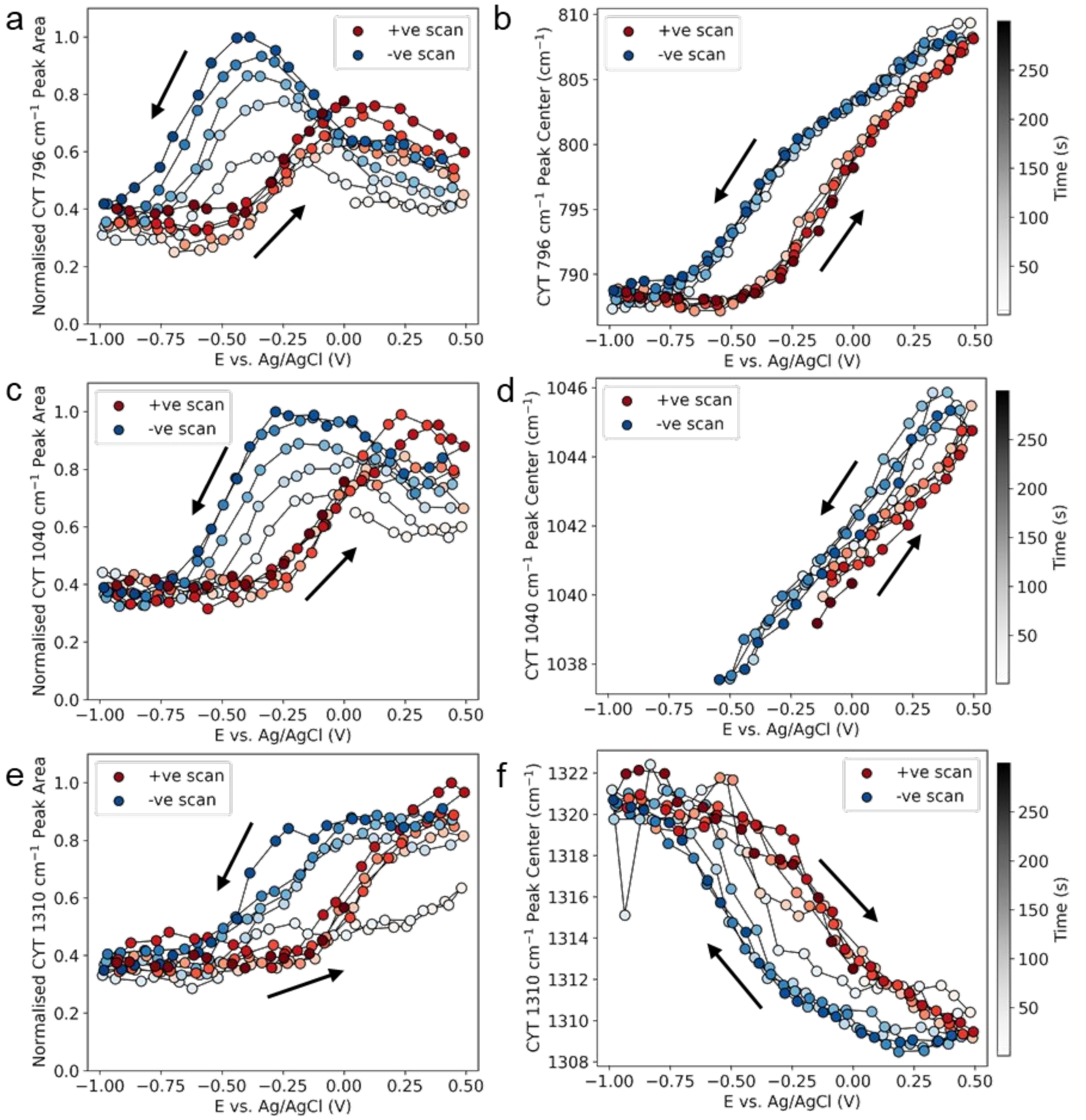

Fig. S13: EC-SERS response of cytosine. (a,c,e) Normalised SERS peak area *vs* applied potential (*vs* Ag/AgCl) for the peaks at (a) 796 cm^{-1} (ring-breathing), (c) 1040 cm^{-1} (ring deformation), and (e) 1310 cm^{-1} (ring stretch C-N).(b,d,f) Peak frequency *vs* applied potential for same peaks as (a,c,e). Marker hue denotes the time elapsed, conditions and cycling as Fig. S11.

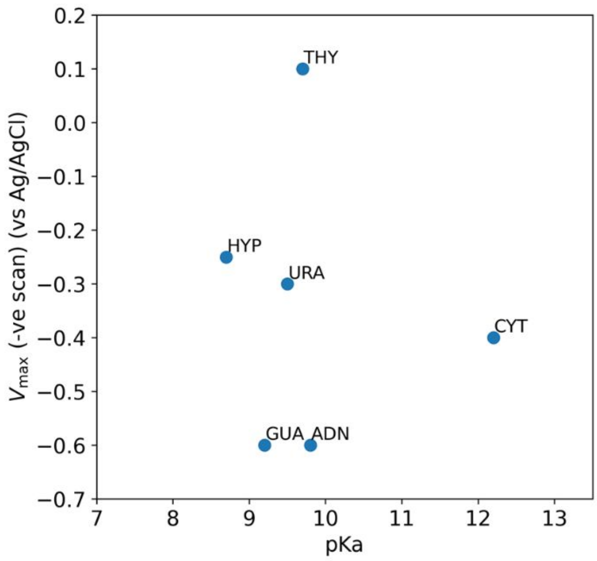

Fig. S14: Relationship between $V_{\mathbf{max}}$ and pKa for different nucleobases. The potential $V_{\max}$ is that at which a maximum SERS signal is recorded during the negative scan for adenine (ADN), cytosine (CYT), guanine (GUA), thymine (THY), uracil (URA), and hypoxanthine (HYP). Conditions and cycling as Fig. 1.

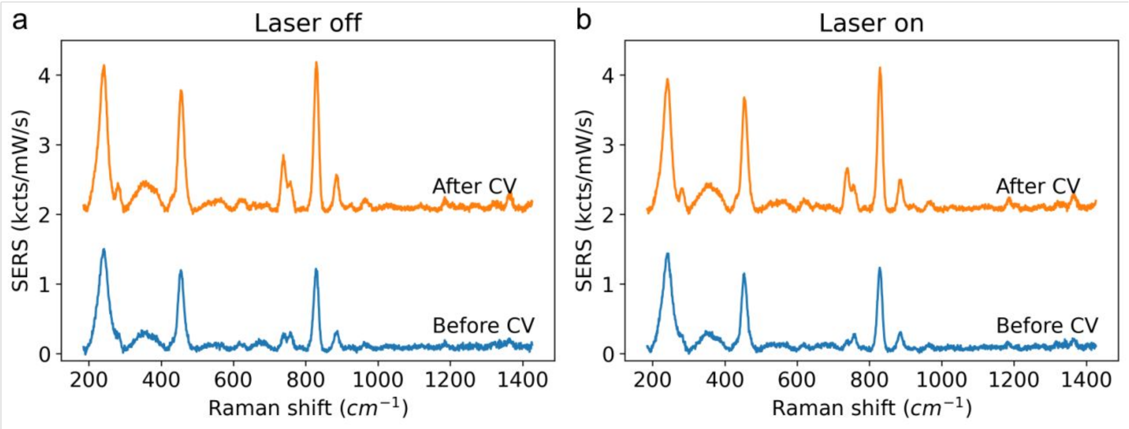

Fig. S15: Effect of laser illumination during potential cycling. Adenine SERS spectra recorded before and after 5 CV cycles performed (a) without and (b) with 785 nm laser exposure (1 mW) during cycling. Conditions and cycling as Fig. 1.

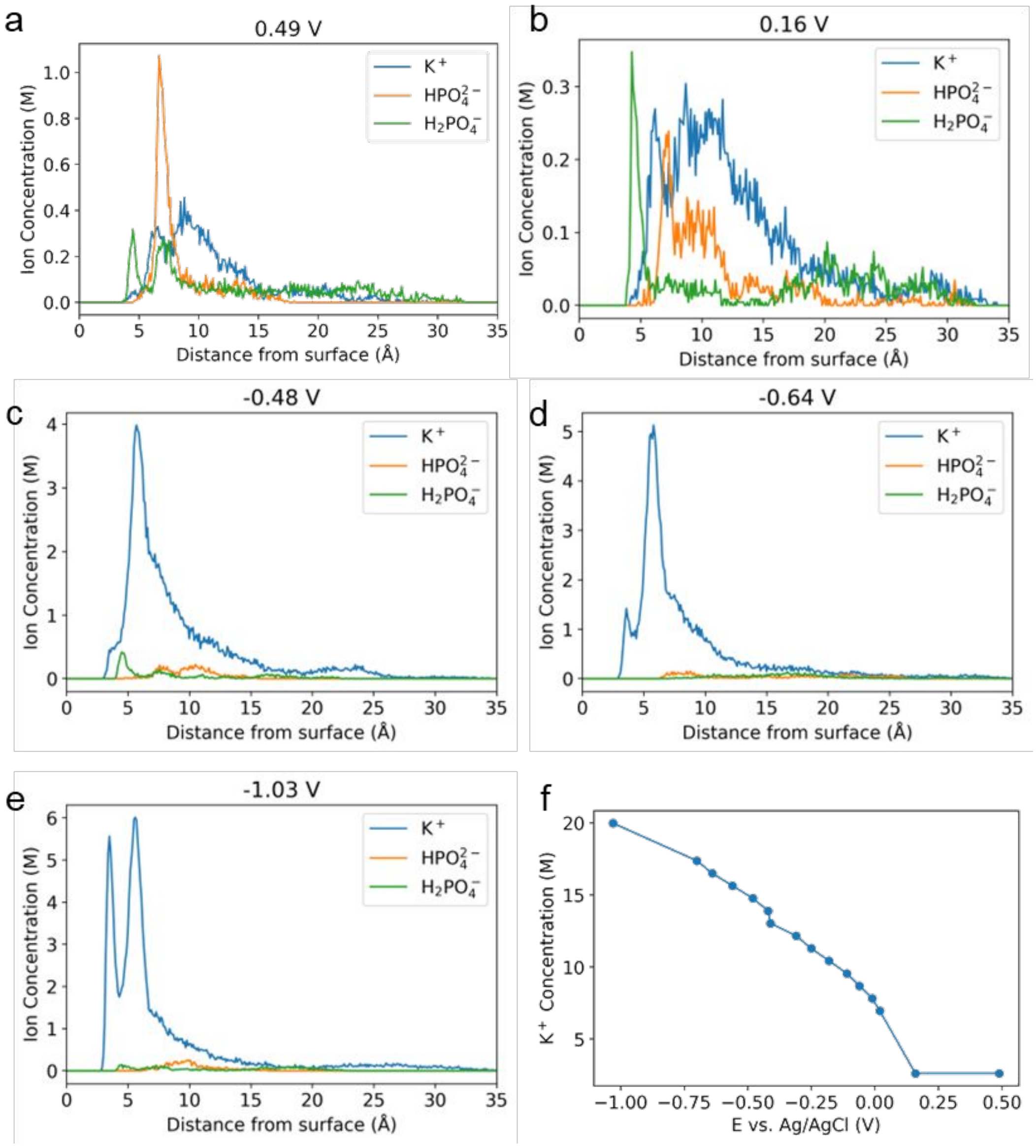

Fig. S16: Ion distributions from QM/MM simulations on planar Au. (a-e) Simulated ion distributions for a 50 mM KPB solution at a Au(111) surface for different applied potentials. (f) Integrated K^+ concentration at the interface at different applied potentials.

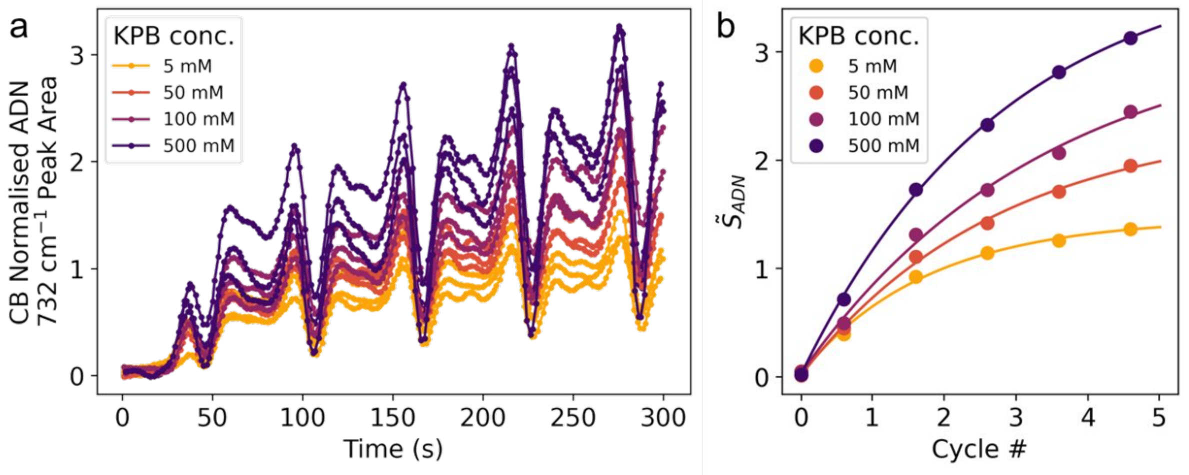

Fig. S17: Effect of buffer concentration on cyclic EC-SERS response. (a) ADN peak area (732 cm^{-1}, normalised by CB[5]) plotted against time for different potassium phosphate buffer (pH 7.0) concentrations. (b) Maximum ADN signal plotted against cycle for the different KPB concentrations. Conditions and cycling as Fig. 1.

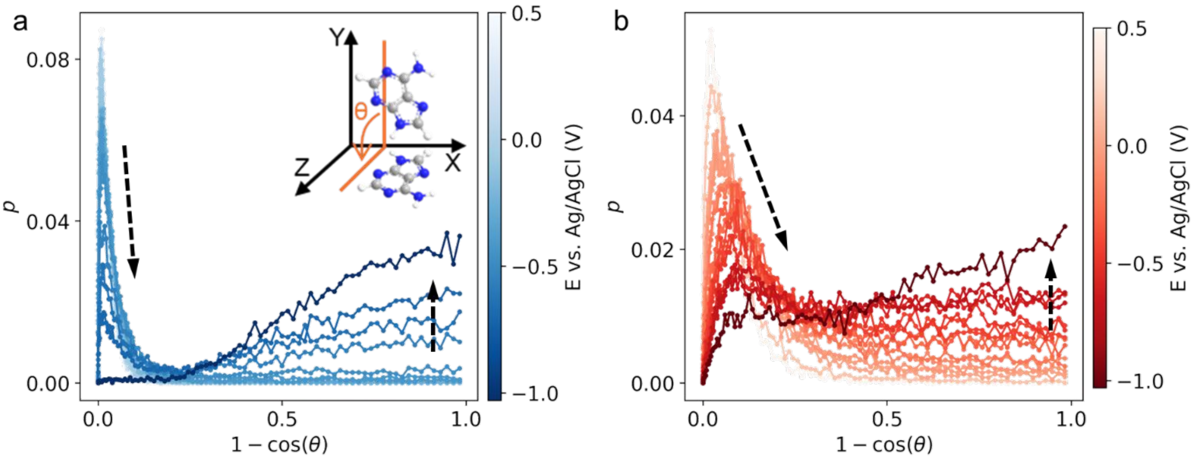

Fig. S18: Simulated orientation of (a) adenine and (b) cytosine on Au (111) at different applied potentials. θ is the angle between the surface normal vector and the z axis.

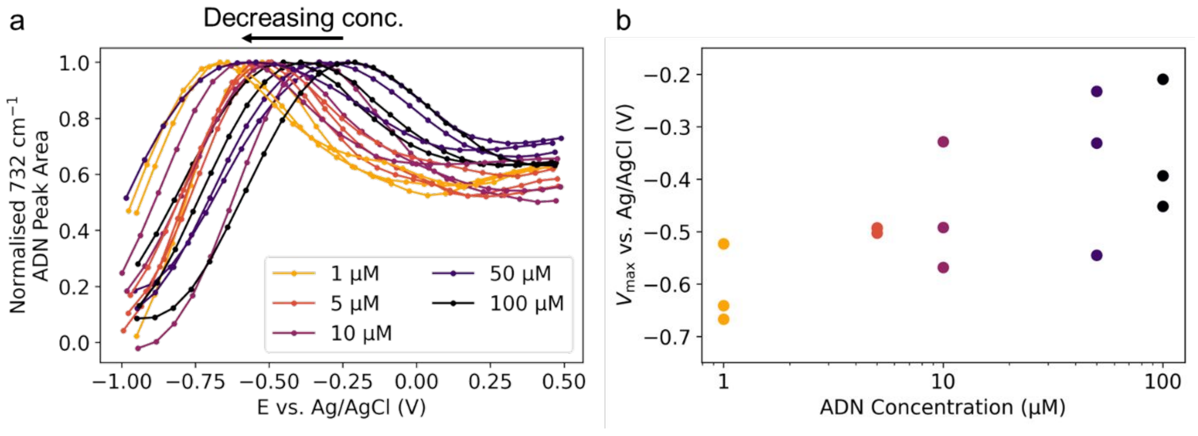

Fig. S19: Comparison of ADN peak signals for different concentrations. (a) Normalised SERS peak area *vs* applied potential (vs. Ag/AgCl) for the peak at 732 cm^{-1} for the negative scan of the first cycle for solutions containing different ADN concentrations. Conditions and cycling as Fig. 1. (b) Potential at which the maximum signal is recorded (V_{max}) plotted against ADN concentration.

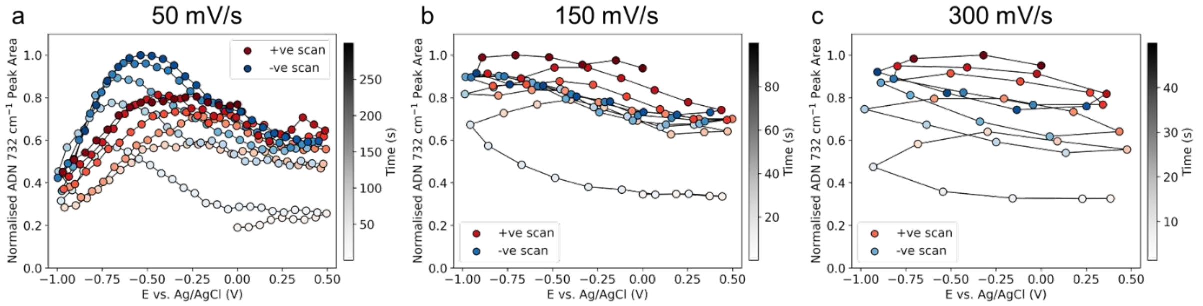

Fig. S20: Comparison of ADN peak signals during EC scans of different rates. Normalised SERS peak area *vs* applied potential (*vs*. Ag/AgCl) for the peak at 732 cm^{-1} (ring-breathing) for an MLagg cycled between +0.5 V and -1 V in 10 µM ADN and 50 mM KPB (pH 7.0) at (a) 50 mV s^{-1}, (b) 150 mV s^{-1}, and 300 mV s^{-1} for 5 cycles. Marker hue denotes the time elapsed.

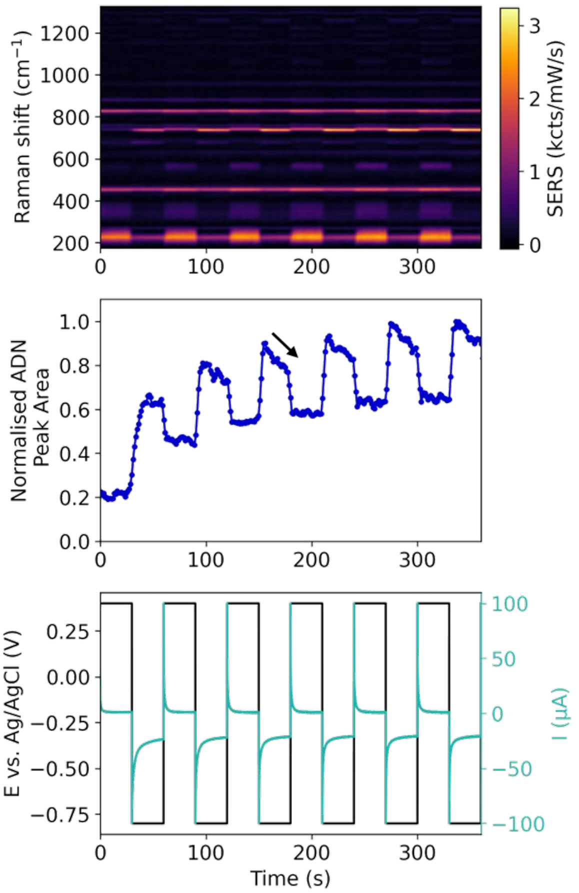

Fig. S21: Effect of applying constant negative potential. Time-series SERS spectra (1 s integration time, 785 nm 1 mW laser) of the MLagg stepped between +0.4 V and -0.8 V in 10 µM adenine (ADN) and 50 mM potassium phosphate buffer (pH 7.0) at 50 mV s^{-1} for 6 cycles. Normalised peak area for ADN peak at 732 cm^{-1} (blue), applied potential (black), and current (cyan) plotted vs time.

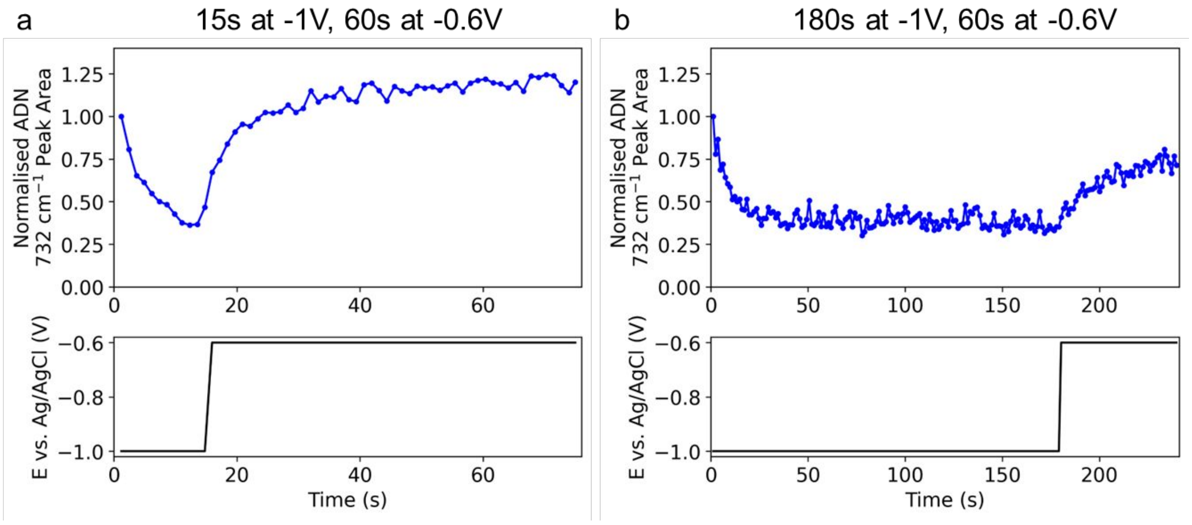

Fig. S22: Adenine desorption with application of constant negative potential: Normalised peak area for adenine (ADN) SERS peak at ~732 cm^{-1} (blue) and applied potential (black) plotted vs time for MLaggs that have been cycled between +0.5 V and -1 V in 10 μM ADN and 50 mM potassium phosphate buffer (pH 7.0) for five cycles, followed by the application of a constant potential of -1V for (a) 15 s and (b) 180 s and then -0.6V for 60s.

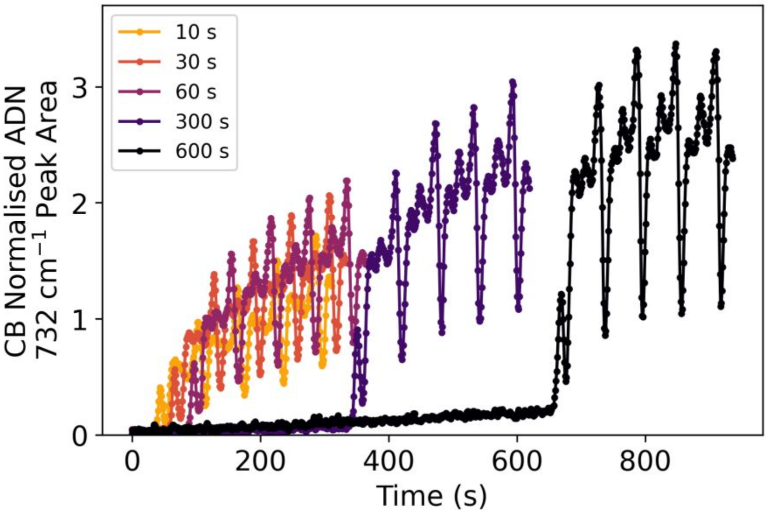

Fig. S23: Effect of waiting at OCP before performing cyclic EC-SERS. ADN peak area (732 cm^{-1}, normalised by CB[5]) for different times spent waiting at OCP (0.2V vs. Ag/AgCl) before cycling the potential. Conditions and cycling as Fig. 1.

S2. Langmuir-Hill model

The concentration series in Figs. 4b and 4c were fitting according to the standard Langmuir-Hill equation:

$$f = \frac{D}{1 + \left(\frac{K_a}{|C|}\right)^n} + b$$

where K_a is the analyte concentration to occupy half of the binding sites (dissociation constant), n is the Hill coefficient, $|C|$ is the analyte concentration, and D and b are constants. These were fitted to Figs. 4b and 4c and the fit coefficients as well as the LoD are detailed in Table S1.

Table S1: Langmuir-Hill fit coefficients for adenine (Fig. 4b) and cytosine (Fig. 4c). The LoD is calculated as 3σ above the noise level.

	Adenine		Cytosine	
Parameters	Initial value	After 5 EC cycles	Initial value	After 5 EC cycles
K_a	13	6.4	6.3	100
n	2.3	1.0	1.8	0.74
D	0.96	3.5	0.55	4.2
b	0.02	0.00	0.03	0.03
LoD (μM)	3.7	0.15	1.7	0.26

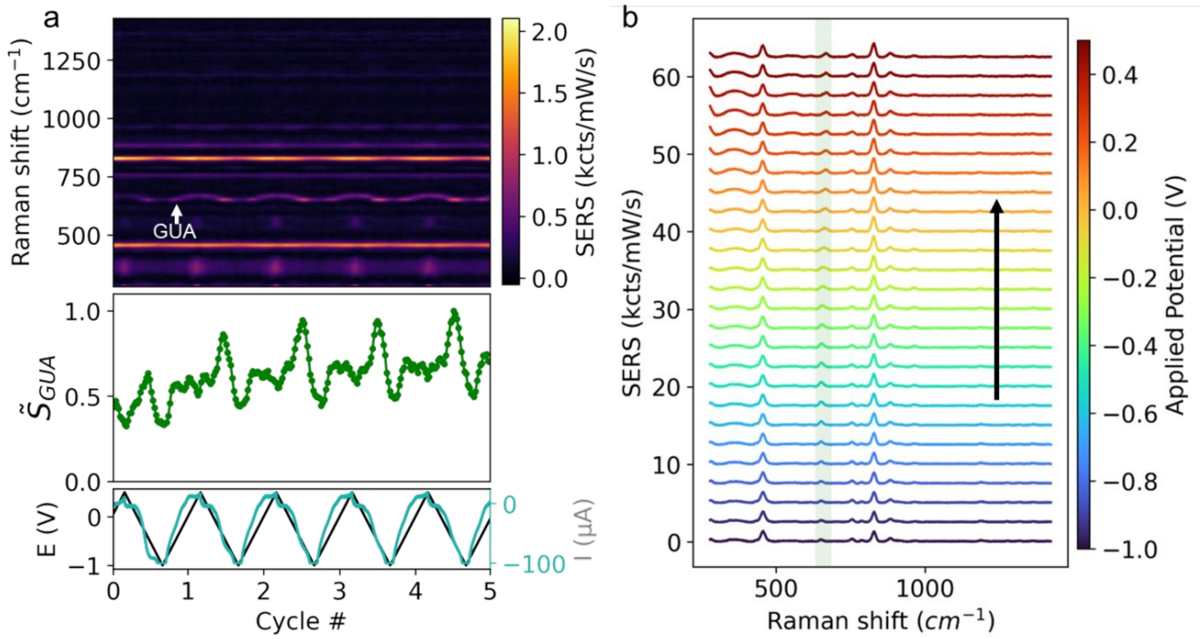

Fig. S24: Cyclic electrochemical SERS of guanine. (a) Time-series SERS spectra (1 s integration time, 785 nm 1 mW laser) of the MLagg cycled between +0.5 V and -1 V in 100 μM guanine (GUA) and 50 mM potassium phosphate buffer (pH 7.0) at 50 mV s^{-1} for 5 cycles. Normalised peak area for GUA peak at 660 cm^{-1} (green), applied potential (black), and current (cyan) plotted vs time. (b) Spectra taken from the first positive cycle from -1 V to +0.5 V. The characteristic GUA peak is shaded in green.

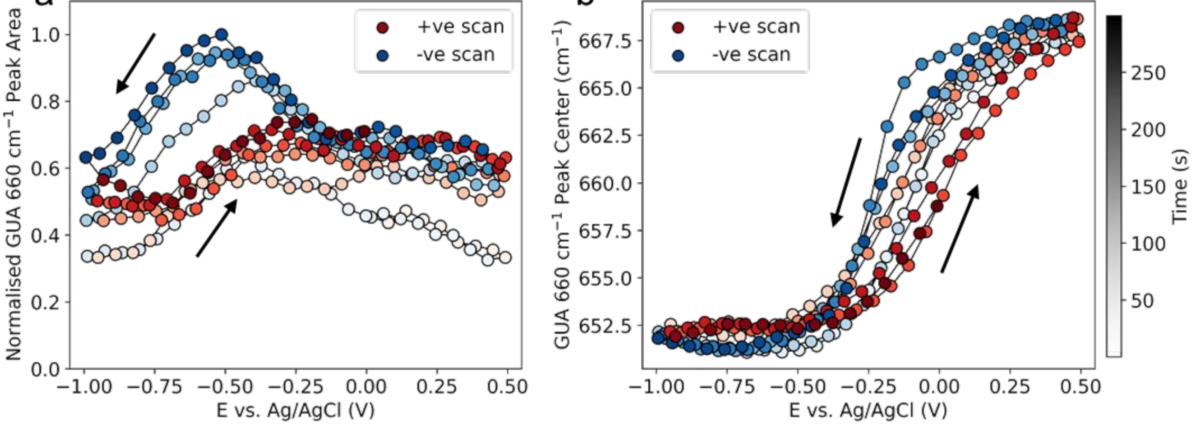

Fig. S25: EC-SERS response of guanine. (a) Normalised SERS peak area *vs* applied potential (*vs* Ag/AgCl) for the peak at 660 cm^{-1} (ring-breathing. (b) Peak frequency *vs* applied potential for the same peaks as (a). Intensity of marker colour denotes the time elapsed, conditions and cycling as Fig. S16.

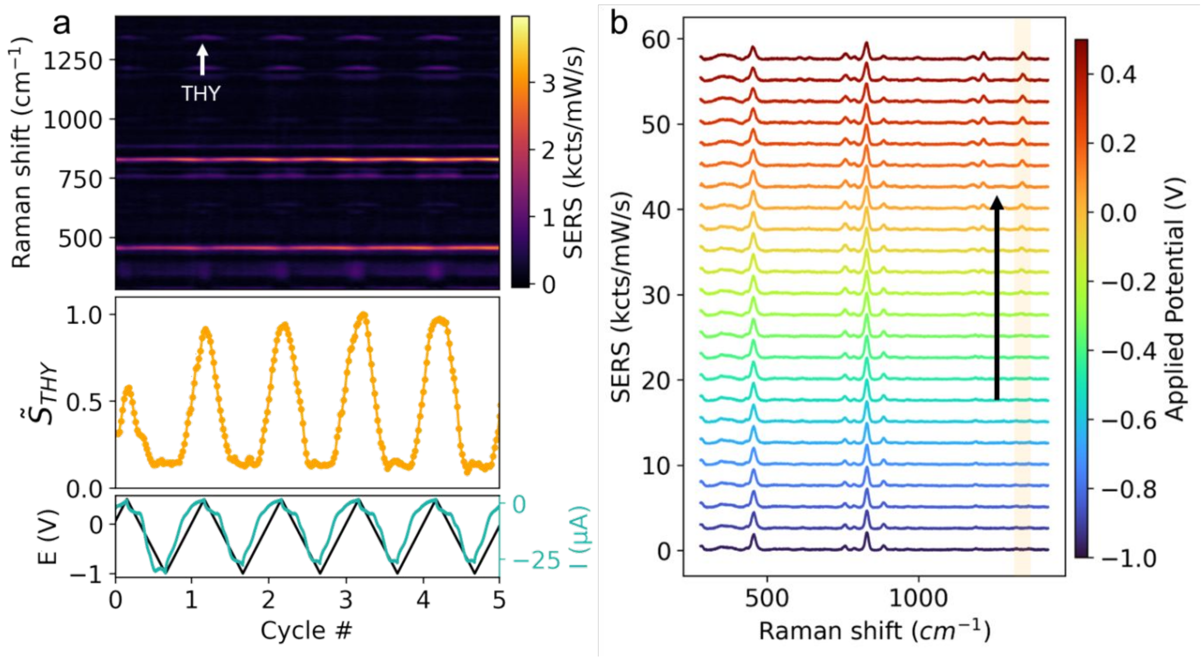

Fig. S26: Cyclic electrochemical SERS of thymine. (a) Time-series SERS spectra (1 s integration time, 785 nm 1 mW laser) of the MLagg cycled between +0.5 V and -1 V in 100 μM thymine (THY) and 50 mM potassium phosphate buffer (pH 7.0) at 50 mV s^{-1} for 5 cycles. Normalised peak area for THY peak at ~1340 cm^{-1} (orange), applied potential (black), and current (cyan) plotted vs time. (b) Spectra taken from the first positive cycle from -1 V to +0.5 V. The characteristic THY peak is shaded in orange.

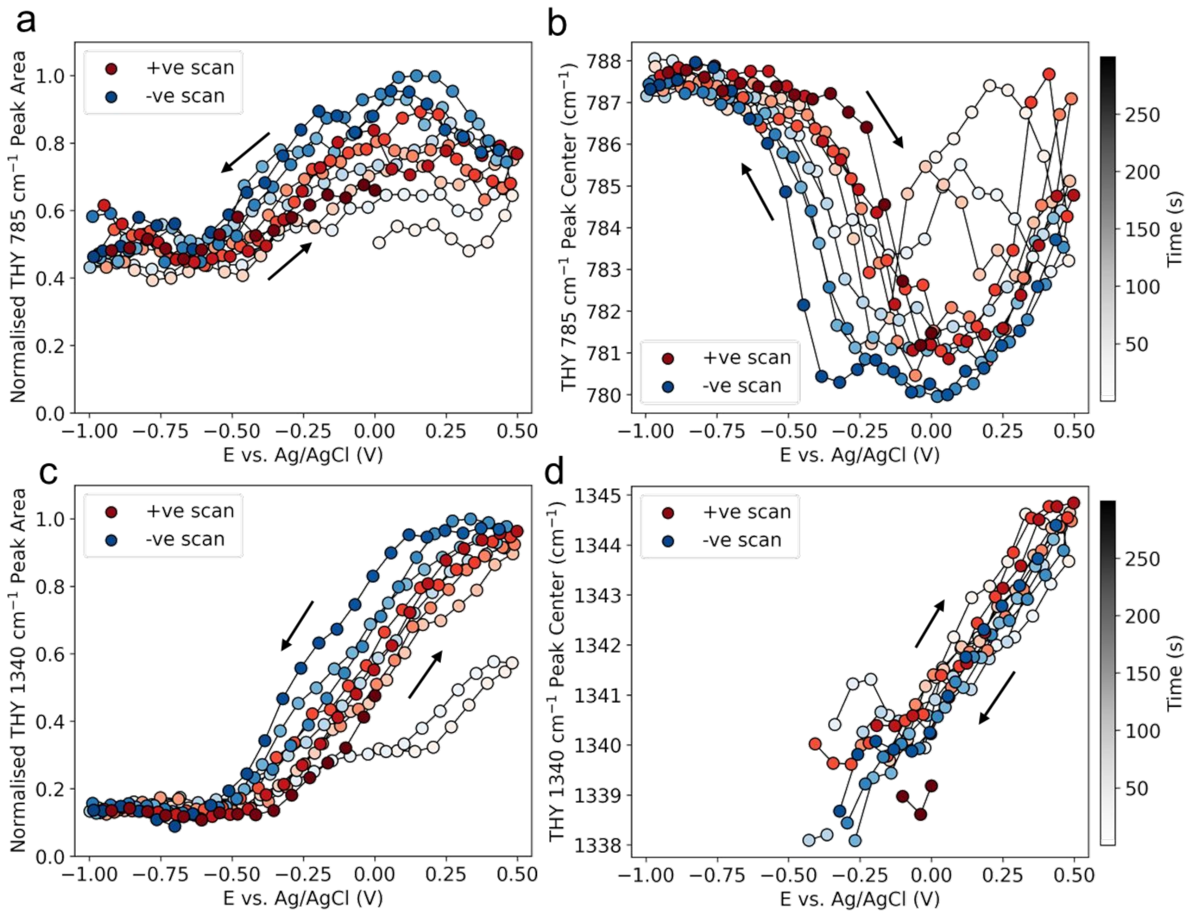

Fig. S27: EC-SERS response of thymine. (a, c) Normalised SERS peak area *vs* applied potential (*vs*. Ag/AgCl) for the peaks at (a) 785 cm^{-1} (ring-breathing) and (c) 1340 cm^{-1} (CH_3 bend). (b, d) Peak frequency *vs* applied potential for the same peaks as (a). Marker hue denotes the time elapsed, conditions and cycling as Fig. S18.

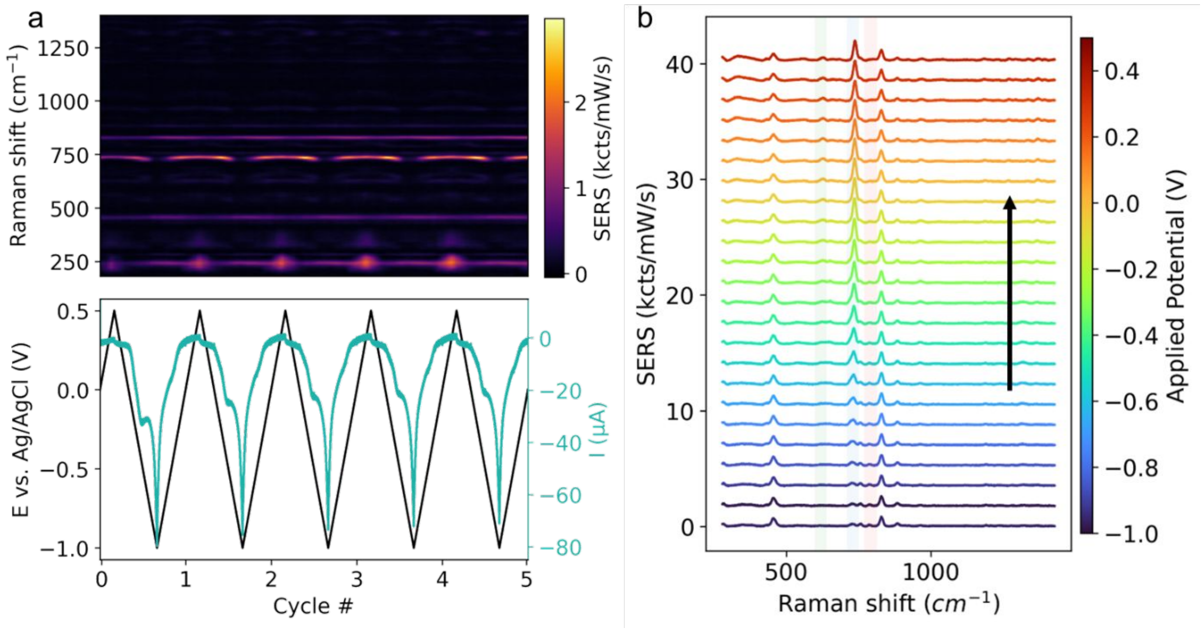

Fig. S28: Cyclic electrochemical SERS of multiple DNA nucleobases. (b) Time-series SERS spectra (1 s integration time, 785 nm 1 mW laser) of the MLagg cycled between +0.5 V and -1 V in 50 µM ADN, 50 µM CYT and 50 µM GUA and 500 mM potassium phosphate buffer (pH 7.0) at 50 mV s^{-1} for 5 cycles. Applied potential (black), and current (cyan) plotted vs time. (b) Spectra taken from the first positive cycle from -1 V to +0.5 V. The characteristic ADN, CYT, and GUA peaks are shaded in blue, red and green respectively.

References

(1) Jang, T.; Shin, S.-J.; Lim, H.-K.; Goddard, W. A. I.; Kim, H. DFT-CES2: Quantum Mechanics Based Embedding for Mean-Field QM/MM of Solid–Liquid Interfaces. *JACS Au* **2025**, *5* (4), 2047–2058.

(2) Grimme, S.; Antony, J.; Ehrlich, S.; Krieg, H. A Consistent and Accurate Ab Initio Parametrization of Density Functional Dispersion Correction (DFT-D) for the 94 Elements H-Pu. *J. Chem. Phys.* **2010**, *132* (15), 154104.

(3) Johnson, E. R.; Becke, A. D. A Post-Hartree-Fock Model of Intermolecular Interactions: Inclusion of Higher-Order Corrections. *J. Chem. Phys.* **2006**, *124* (17), 174104.

(4) Giannozzi, P.; Baroni, S.; Bonini, N.; Calandra, M.; Car, R.; Cavazzoni, C.; Ceresoli, D.; Chiarotti, G. L.; Cococcioni, M.; Dabo, I.; Dal Corso, A.; de Gironcoli, S.; Fabris, S.; Fratesi, G.; Gebauer, R.; Gerstmann, U.; Gougoussis, C.; Kokalj, A.; Lazzeri, M.; Martin-Samos, L.; Marzari, N.; Mauri, F.; Mazzarello, R.; Paolini, S.; Pasquarello, A.; Paulatto, L.; Sbraccia, C.; Scandolo, S.; Sclauzero, G.; Seitsonen, A. P.; Smogunov, A.; Umari, P.; Wentzcovitch, R. M. QUANTUM ESPRESSO: A Modular and Open-Source Software Project for Quantum Simulations of Materials. *J. Phys. Condens. Matter* **2009**, *21* (39), 395502.

(5) Plimpton, S. Fast Parallel Algorithms for Short-Range Molecular Dynamics. *J. Comput. Phys.* **1995**, *117* (1), 1–19.

(6) Jang, T.; Paik, D.; Shin, S.; Kim, H. Density Functional Theory in Classical Explicit Solvents: Mean-Field QM/MM Method for Simulating Solid–Liquid Interfaces. *Bull. Korean Chem. Soc.* **2022**, *43* (4), 476–483.
